# Lifetime Physical Loading and Magnetic Resonance‐Derived Intervertebral Disc Health in Adults With Chronic Low Back Pain: A Cross‐Sectional Study

**DOI:** 10.1002/jsp2.70186

**Published:** 2026-05-15

**Authors:** Claire L. Samanna, Christopher Neason, Scott D. Tagliaferri, Ulrike H. Mitchell, Michelle Caldwell, Ali Imran, Hana Rae Nez, Daniel L. Belavý, Clint T. Miller, David Scott, Niamh L. Mundell, Paul Buntine, Patrick J. Owen

**Affiliations:** ^1^ Eastern Health Clinical School Monash University Melbourne Victoria Australia; ^2^ Institute of Physical Activity and Nutrition, School of Exercise and Nutrition Sciences Deakin University Geelong Victoria Australia; ^3^ Sport, Performance, and Nutrition Research Group, School of Allied Health, Human Services and Sport La Trobe University Melbourne Australia; ^4^ Orygen Parkville Victoria Australia; ^5^ Centre for Youth Mental Health University of Melbourne Melbourne Victoria Australia; ^6^ Department of Exercise Sciences Brigham Young University Provo Utah USA; ^7^ Deakin University, School of Medicine Geelong Victoria Australia; ^8^ Integrated Pain Specialists South Jordan Utah USA; ^9^ Division of Physiotherapy, Department of Applied Health Sciences Bochum University of Applied Sciences Bochum Germany; ^10^ School of Clinical Sciences at Monash Health Monash University Clayton Victoria Australia; ^11^ Eastern Health Emergency Medicine Program Melbourne Victoria Australia

**Keywords:** back pain, lifespan exercise, lumbar imaging, spinal disc, spine, sports

## Abstract

**Background:**

The intervertebral disc (IVD) is a mechanosensitive structure influenced by physical loading (physical activity/sport/exercise); however, the optimal type and parameters of physical loading for IVD health remain unclear. The Bone Physical Activity Questionnaire is validated to predict bone mineral density from lifetime exposure to physical loading; therefore, we explored the association between lifetime physical loading and IVD health.

**Methods:**

This cross‐sectional study recruited 40 individuals with chronic low back pain. Outcomes were magnetic resonance imaging‐derived IVD measures reported as both averages and across individual spinal levels T11/T12–L5/S1, including T2 (ms, measure of hydration), height‐to‐vertebral‐body ratio, nucleus‐to‐annulus signal intensity ratio, volume (cm^3^), and Pfirrmann grade (0–5 points). Multiple linear regression examined lifetime physical loading and IVD health while controlling for significant covariates (age, body mass index, current and past occupational sitting, and physical labour).

**Results:**

Lifetime bone‐related physical loading was not associated with IVD health across average spine values, except nucleus‐to‐annulus signal intensity ratio, which was negatively associated (*β* [95% CI]: −0.00 [−0.01, −0.00], *p* = 0.030). At L5/S1, IVD T2 (−0.30 [−0.56, −0.05], *p* = 0.022) and nucleus‐to‐annulus signal intensity (−0.02 [−0.03, −0.01], *p* = 0.007) were negatively associated, and Pfirrmann grade (0.02 [0.01, 0.03], *p* = 0.006) was positively associated with physical loading.

**Conclusions:**

Physical activity loading necessary for improved IVD health appears to differ from loading previously reported for optimal bone health. Specifically, lifetime activity levels considered beneficial for bone were associated with less favourable L5/S1 IVD characteristics, highlighting distinct loading requirements across these tissues. Future studies could develop a questionnaire that captures optimal physical loading for IVD health, such as moderate physical loading.

## Graphical abstract

Final multiple linear regression model: heat maps showing Bone‐specific Physical Activity Questionnaire score associations with T2 values in five intervertebral disc (IVD) regions with statistically significant covariates controlled for at individual spinal levels.

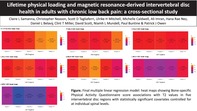

## 1 Introduction

Low back pain affects over half a billion individuals globally and is the leading cause of years lived with disability [1, 2]. In 2022–2023, low back pain cost the Australian healthcare system $3.9 billion [3] and is the primary reason for early retirement [4]. One viable inroad to this global health issue is understanding the link between poor intervertebral disc (IVD) health and low back pain [5]. Although IVD degenerative processes occur naturally with ageing [6], signs of poor IVD health are up to eight times more likely in individuals experiencing low back pain [5]. A healthy IVD consists of three distinct parts: (a) the vertebral endplate, which facilitates diffusion of nutrients and water; (b) the nucleus pulposus, a highly hydrated inner core that distributes spinal loads; and (c) the annulus fibrosus, a fibrous and structured outer layer that maintains the position of the nucleus while supporting controlled spinal movement [7]. Poor IVD health is characterised by reduced hydration, increased fibrous cartilage, and diminished distinction between the nucleus and annulus. These degenerative changes lower the IVD hydrostatic pressure, height, and volume, ultimately impairing its capacity to distribute spinal loads effectively [8]. Therefore, understanding the long‐term factors that influence IVD health in individuals with chronic low back pain warrants attention.

Several modifiable risk factors influence poor IVD health, such as diabetes (type two) [9], smoking [10], obesity [11], and excessive sedentary time [12]. Inactivity or a lack of physical loading (exercise/sport/physical activity) is associated with worse IVD health [13]; however, the relationship between physical loading and markers of IVD health is uncertain. For example, cross‐sectional studies report a higher prevalence of poor IVD health among athletes participating in sports with high compressive loading (weightlifting [14]) and repetitive end‐range loading (fast‐pace bowling in cricket [15] and elite gymnastics [16]). Another study showed poorer IVD health among international‐level gymnastics athletes compared to amateur and national gymnastic athletes [17], suggesting that higher loading volumes, rather than moderate ones, may drive the poorer IVD health. In contrast, other cross‐sectional studies have shown that different sports, such as running [18, 19], rowing [20], and martial arts [21], were associated with healthier IVDs compared with controls. In particular, a dose–response relationship was observed, where individuals running more than 50 kms per week showed greater IVD health than those running 20–40 kms per week [18]. This conflicts with the aforementioned gymnastics study and suggests that the type of physical loading matters; however, both studies are limited to a single sport. Further, these cross‐sectional data alone provide little evidence on exposure over longer time frames (decades) and in non‐athletic populations. A monozygotic twin study with contrasting lifetime exercise history assessed spinal health at mid‐life (mean age: 50 years) and found that lifetime exposure to power‐based sports (weightlifting) was associated with poorer IVD health, whereas no differences were observed for endurance‐based sports (running and cross‐country skiing) [22]. Nonetheless, these results are limited to one study, based on binary separation of two types of physical loading, and recall only to 12 years old, missing vital physical loading exposure in early childhood. Hence, there are several gaps in understanding how lifetime exposure of physical loading influences the IVD in adulthood.

The Bone Physical Activity Questionnaire (BPAQ) quantifies physical loading over a lifespan. The BPAQ is based on the concept that higher bone loading through sport, physical activity, and exercise (i.e., physical loading), especially in childhood [23], leads to greater bone mineral density in adulthood. The BPAQ has been validated to calculate hip [24], femoral neck [24], and whole body [25] bone mineral density via ground reaction forces accelerometry across 19 physical activities [26]. Current data suggest that fast walking and slow jogging may be the most beneficial loading for IVD health [18], although evidence is not well established. Therefore, the current study used the BPAQ to quantify lifetime physical loading exposure and assess its potential impact on the IVD, to determine if the IVD adapts to loading like bone. Applying the BPAQ in adult populations with chronic low back pain may provide insight into whether the physical loading effects observed in bone health are also observed in IVD health in a population that is more likely to have degenerated IVDs. Therefore, the aim of this study was to assess whether magnetic resonance‐derived IVD measures are associated with BPAQ scores across the average spine and individual spinal levels from T11/T12‐L5/S1 in individuals with chronic low back pain. The secondary aim was to explore the role of potential IVD covariates with BPAQ scores to test the robustness of our findings. Finally, we examined whether self‐reported pain and disability were associated with BPAQ scores to understand if there was a relationship between physical loading and low back pain in this clinical population. These findings may help guide the design of an alternate and easily administered questionnaire to assess IVD health in relation to lifetime physical loading exposure.

## 2 Methods

### 2.1 Study Design

This cross‐sectional study is a secondary analysis of baseline data from the ASTEROID randomised controlled trial, reported per the STROBE checklist (Supporting Information A). The protocol [27] and primary outcomes [28] are detailed elsewhere. The study was registered with the Australian New Zealand Clinical Trials Registry (ACTRN12622001276741, date registered: 29/09/2022) and approved by the Deakin University Human Research Ethics Committee review board (ID: 2022‐162, date registered 29/09/2022). In line with the Declaration of Helsinki, all participants provided written informed consent prior to participation.

### 2.2 Setting

All baseline data were collected from December 2022 to February 2023 at a commercial radiological imaging facility in Melbourne, Victoria (Imaging@Olympic Park).

### 2.3 Participants

Participants were recruited via social media advertising in the Melbourne metropolitan region. Expressions of interest were linked to the study website, where researchers (CLS, CN) screened individuals for eligibility according to the following criteria: (a) aged 18–45 years and (b) reported chronic non‐specific low back pain defined as pain located between the costal margin and above the inferior gluteal fold with pain on most days of the week (> 3 days), for greater than 3 months. Exclusion criteria were: (a) specific low back pain (fracture, cauda equina syndrome, infection, cancer, spondyloarthropathies); (b) low back pain secondary to trauma (car accident or fall); (c) previous spinal surgery; (d) structural scoliosis requiring surgical consultation; (e) current radiculopathy defined as leg pain greater than low back pain; (f) non‐English speaking; (g) recent pregnancy (< 1 year) or currently lactating; (h) current or prior elite athlete (member of a national sport team or the Australian or state institutes of sports) [29]; (i) absolute contraindications for exercise training [30]; (j) high‐risk category via the Adult Pre‐Exercise Screening System [31]; (k) recent participation in running or sport involving running (> 1 session once per month); (l) recent lower limb injury (< 6 weeks) [32]; and (m) no access to a smartphone with cellular internet connection.

### 2.4 Data Sources/Measurements

#### 2.4.1 Magnetic Resonance Imaging Protocol

Lumbar IVD data were obtained from baseline magnetic resonance images acquired prior to randomisation in the larger trial. Sequences and specifications used in magnetic resonance imaging are reported in detail in Table 1. To image IVDs, we used a 3 T Phillips (Best, Netherlands; software release 5.72021‐10‐04) magnetic resonance imaging machine with spinal coils, a posterior coil, and torso coil array anteriorly. All scans captured a sagittal plane view of the entire lumbar spine from left to right at varying echo times. For consistency and to reduce the impact of diurnal changes [33], prior to magnetic resonance imaging, participants were advised to: avoid exercise on the same day, schedule the scan time at least 4 h after waking, and sit for 20 min immediately before the scan. Sagittal spin‐echo multi‐echo sequences with eight echo times were used to quantify average IVD T2, height, and volume. Sagittal T2 Sag spin‐echo sequences with one echo‐time were used to quantify nucleus‐to‐annulus signal intensity ratio and Pfirrmann grade. MDIXON SAG TE sequences with two echo‐times were used to quantify vertebral body height.

**TABLE 1:** Magnetic resonance imaging sequences and specifications for lumbar spine.

	Sagittal spin‐echo multi echo	T2 Sag spin‐echo	mDIXON SAG TE
Echo times	15.75, 36.75, 57.75, 78.75, 99.75, 120.75, 141.75 and 162.75 ms	70.0 ms	2.372 ms and 1.186 ms
Slices	12 sagittal	15 sagittal	80 sagittal slices—4 × 20 acquisitions
Slice thickness	3.5 mm	3.5 mm	3.0 mm
Interslice distance	1.0 mm	1.0 mm	0.0 mm
Spacing	4.5 mm	4.5 mm	3.0 mm
Repetition time	2000 ms	2068 ms	17.5065 ms
Field‐of‐view (mm)	634 mm × 669 mm × 54 mm	400 mm × 400 mm × 67.5 mm	400 mm × 400 mm × 240 mm
Field‐of‐view matrix (pixels)	704 × 704 pixels	768 × 768 pixels	512 × 512 pixels
Resolution	0.86 mm^2^/pixel	0.27 mm^2^/pixel	0.61 mm^2^/pixel
Voxel size	0.90 mm × 0.95 mm	0.52 mm × 0.52 mm	0.78 mm × 0.78 mm

*Note:* Magnetic resonance imaging machine: 3TPhillips Magnetic resonance imaging machine (Best, Netherlands; software release 5.72021‐10‐04) with posterior coil and torso coil array anteriorly.

#### 2.4.2 Magnetic Resonance Imaging Data Processing

All magnetic resonance data were obtained in Digital Imaging and Communications in Medicine format and processed in ImageJ (version 1.53 t, https://imagej.net/ij/). Three researchers (CLS, UHM, AI) independently traced one scan sequence each for consistency, using established methods that have demonstrated excellent reliability within the same research team (ICC = 0.98) [34]. The methods used a semi‐automated, point‐guided segmentation approach in which the tracer placed a series of points around the IVD boundary. The software connects these points to form a closed boundary, which could be manually adjusted after generation to refine the final segmentation. These methods were applied with all tracers blinded to participant characteristics and lifetime physical activity exposure through the use of randomly assigned image codes, consistent with previous methodological practice [34].

Before starting, all three researchers (CLS, UHM, AI) traced five pilot scans for review by a researcher experienced in these tracing methods (SDT). This process involved, first, locating and documenting the middle sagittal slice identified by the spinous process. Second, the region of interest was manually selected on all visually distinct slices where the borders of the IVD or vertebral body were clearly intact. Slices in which the borders or sections of the borders were not visible were not traced (i.e., slices at the start or end of the 12‐slice set). The same coordinates were extracted across all echo times using a custom‐made plugin (“ROI Analyzer”; https://github.com/tjrantal/RoiAnalyzer and https://sites.google.com/site/daniellbelavy/home/roianalyser). Further data processing was conducted in the R statistical environment (version 4.4.1, https://www.r‐project.org/).

#### 2.4.3 Magnetic Resonance Imaging Outcomes

**FIGURE 1:**
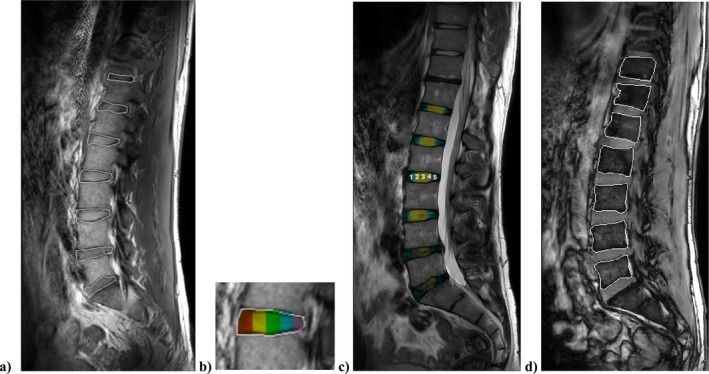
(a) Sagittal spin‐echo multi‐echo image showing lumbar intervertebral discs (IVD; L5/S1 to T11/T12) with the traced IVD region of interest in ImageJ, used for T2, height, and volume calculations. (b) Five anterior–posterior subregions used to derive regional T2 values (anterior annulus, anterior nucleus, central nucleus, posterior nucleus, and posterior annulus). Colours are shown for illustration only; actual subregions were generated automatically using equidistant segmentation. (c) T2‐weighted sagittal image used for nucleus‐to‐annulus signal intensity ratio calculations (mean of the middle three regions divided by the mean of the outer two regions). (d) Sagittal mDixon TE image with traced vertebral body region of interest used to calculate vertebral body height for the IVD‐to‐vertebral body ratio.

The following magnetic resonance imaging‐derived outcomes were generated and are described below (see Figure 1).

1. IVD T2 values (ms): T2 relaxation times were calculated from the spin‐echo multi‐echo sequences by applying a linear fit to the natural logarithm of the signal intensity across eight echo times (15.75, 36.75, 57.75, 78.75, 99.75, 120.75, 141.75, and 162.75 ms). We assumed an exponential decay of the MRI signal with echo time and estimated T2 by fitting a straight line to the log signal across all eight echoes. All eight echoes were included without explicit stimulated‐echo suppression. Analyses used data from 12 slices with a thickness of 3.5 mm. For each lumbar level, T2 values from all available slices were averaged to obtain a single level‐specific T2 value. In addition, regional T2 values were derived from five equidistant anterior–posterior regions that were automatically segmented along the IVD width. These five regions correspond to the anterior annulus, anterior nucleus, central nucleus, posterior nucleus, and posterior annulus.
2. IVD morphometry: The IVD height (mm), width (mm), and area (mm^2^) were obtained from 2D spin‐echo multi‐echo sequences on each traced slice. Height was defined as the mean straight‐line distance between the opposing endplate boundaries sampled across the entire IVD on each slice (i.e., an average taken over the whole IVD rather than a single central measurement). Width was measured as the straight‐line distance from the anterior to posterior IVD margins on the same slice. For each lumbar level, average values for each slice were averaged again, across all available slices to yield one level‐specific height, width, and area. From these, the following metrics were derived:
  1. IVD height‐to‐vertebral body ratio: IVD height was normalised relative to vertebral body height by dividing the average IVD height by the average vertebral body height at each lumbar level to calculate a dimensionless ratio.
  2. IVD volume (cm^3^): Volume was calculated as the sum of traced slice areas multiplied by the effective slice spacing (3.5 mm slice thickness + 1 mm interslice gap = 4.5 mm), converting to cubic centimetres.
3. Vertebral body morphometry: The average vertebral body height (mm) was extracted from the 2D mDIXON sagittal TE sequences at each available slice. For each lumbar level, the height values from all slices were then averaged to obtain a single vertebral body height measurement. The IVD height‐to‐vertebral body ratio was calculated by dividing the average IVD height by the average vertebral body height at each lumbar level.
4. IVD nucleus‐to‐annulus ratio: The average signal intensity at five equidistant anterior–posterior regions of the IVD was extracted from the 2D T2‐weighted sagittal spin‐echo images. For each lumbar level, the regional averages from all slices were then averaged to obtain a single value per region. The nucleus‐to‐annulus ratio was calculated by dividing the mean signal intensity of regions two, three, and four (nucleus) by the mean of regions one and five (annulus) for each lumbar level.
5. Pfirrmann grade: The mid‐sagittal slice from Sagittal T2 Sag spin‐echo sequences, where the spinous process was most visible, was visually assessed by two independent clinical researchers (UHM, HRN), trained in Pfirrmann grading [35]. Any conflicts were resolved via discussion.

All IVD outcomes were analysed as (1) participant‐level averages across all measured spinal levels and (2) for each IVD individually.

### 2.5 Additional Variables

All other outcome measures were assessed via electronic questionnaires (REDcap, Nashville, United States of America) [36]. Participant characteristics were recorded, including sociodemographic details, low back pain history, smoking status, and habitual physical activity, measured using the International Physical Activity Questionnaire (IPAQ).

#### 2.5.1 Lifetime Physical Loading

The BPAQ was a retrospective assessment used to quantify loading impact on bone via recall of lifetime physical activity levels [26]. The self‐administered questionnaire consists of two parts, past and current physical loading. Past loading is quantified by physical loading type and years participated from age 1 year‐old to 12‐months prior to the present time. Current physical loading considers physical loading performed over the last 12 months and the frequency of days. The total BPAQ score is a combination of past and current BPAQ scores, reflecting lifetime habitual loading exposures. All physical loading was assigned a positive value, with greater ground reaction force equating to larger values; hence the higher the BPAQ score, the greater the physical loading. BPAQ scores were calculated by one independent researcher (MC) using the BPAQ online calculator tool and reviewed by another (CLS).

#### 2.5.2 Pain Intensity (Visual Analogue Scale)

The three‐item visual analogue scale was used to quantify current, average (in last week) and worst low back pain intensity [37]. Participants were asked to report their pain intensity by marking a line on a 0–100 points scale, where zero was considered ‘no pain’ and 100 ‘the worst pain imaginable’ [38]. The visual analogue scale has demonstrated high test–retest reliability in chronic musculoskeletal pain (*r* = 0.87) [39].

#### 2.5.3 Disability (Oswestry Disability Index)

The Oswestry disability index measured average disability (inability to complete activities of daily living in the last week) [40]. The 10‐item questionnaire identifies limitations on activities of daily living and questions are rated from 0 to 5, where higher scores indicate greater disability [40]. The Oswestry Disability Index has demonstrated good test–retest reliability (ICC = 0.87) [41].

#### 2.5.4 Covariates

Variables considered as potential covariates for IVD health were collected. Demographic characteristics included age (years) [6], biological sex [42], and body mass index [kg/m^2^] [43]. Occupational physical loading, such as occupational heavy labour is a risk factor for IVD degeneration [44], therefore the validated four‐item Occupational Sitting and Physical Activity Questionnaire was used to assess current and past hours exposed to occupational physical loading.

### 2.6 Bias

All magnetic resonance images were allocated a random code, separate from the participant number by an independent researcher, thereby blinding MRIs for the tracing steps. BPAQ scores were not calculated and reviewed until after the completion of magnetic resonance data processing. Confounding bias was addressed by stratifying outcomes by biological sex in the primary analysis and controlling for potential covariations in multiple linear regression models (age, body mass index, and current‐ and past‐occupational sitting and physical labour exposure).

### 2.7 Study Size

A convenience sample of the total 40 participants from the ASTEROID randomised control trial at the baseline time point was used.

### 2.8 Quantitative Variables

All magnetic resonance‐derived IVD and vertebral body measures were continuous outcomes (apart from Pfirrmann grade) and set up as long data for each spinal level from T11/T12 to L5/S1. All covariate outcomes were treated as continuous variables.

### 2.9 Statistical Methods

All analyses were conducted using Stata (v17, StataCorp, College Station, United States of America). Separate simple linear regressions were conducted to examine the association between lifetime physical loading (BPAQ score) and magnetic resonance‐derived IVD outcomes across all participants. Analyses were conducted separately for each individual spinal level to avoid within‐subject correlations across disc levels. Additional analyses were performed using average spine values. These associations were also explored stratified by biological sex using pooled IVDs data from T11/T12 to L5/S1. Finally, the association between the BPAQ score and IVD T2 was assessed across five anterior‐to‐posterior IVD regions within each individual spinal level. Potential covariates for IVD health (age, body mass index, current‐ and past‐occupational sitting and physical labour exposure) were examined via linear regression models with all magnetic resonance outcomes and significant contributors added to the final multiple linear regression models. No missing data were identified. No further subgrouping was applied. An *α* of 0.05 was adopted for all analyses. Sensitivity analyses applied the Benjamini–Hochberg false discovery rate correction to groups analysed across multiple outcomes or IVD regions, to control the heightened risk of Type I errors (i.e., at each aggregate lumbar level or for average spine values) [45]. All statistical analysis and reporting are consistent with the CHecklist for statistical Assessment of Medical Papers (CHAMP) statement [46].

## 3 Results

### 3.1 Participants

**FIGURE 2:**
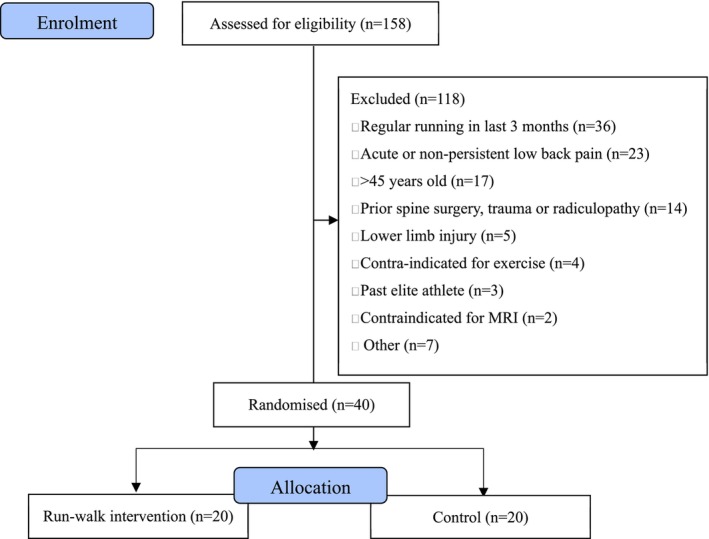
Consort diagram of study flow. *N* = 1 control participant did not complete MRI testing at 6 and 12 weeks due to schedule conflicts.

Participant recruitment was conducted from October 2022 to January 2023 until a sample of 40 participants was obtained (female: *n* = 20, male: *n* = 20). In total, 322 individuals registered interest in the study, with 155 individuals screened for eligibility, from which 61% did not meet the inclusion criteria, and 14% declined participation (Figure 2). Data were collected between December 2022 and March 2023.

### 3.2 Outcome and Descriptive Data

All participant data (*n* = 40) were analysed. Mean (SD) age was 32.9 (6.1), current low back pain duration was 4.1 (4.5) years, first low back pain onset was 6.9 (5.8) years, average low back pain intensity (0–100 scale) was 39.7 (20.8) points, and disability (0–100 scale) was 22.0 (9.0) points. Further demographic characteristics stratified by sex were reported in Table 2.

**TABLE 2:** Descriptive characteristics of participants at baseline.

	Male (*n* = 20)	Female (*n* = 20)
Age, years	35.15 (5.85)	30.55 (5.43)
Body mass index	28.67 (4.33)	29.90 (8.95)
Current low back pain duration, years	4.89 (5.63)	3.22 (2.88)
Average pain, visual analogue scale (0–100)	37.4 (22.75)	42.05 (18.56)
Disability, Oswestry disability index (0–100)	19.6 (9.12)	23.84 (7.89)
Average Pfirrmann grade (1–5)	3.06 (0.59)	2.95 (0.60)
Total bone physical activity loading score	16.97 (17.66)	12.00 (13.39)
Habitual physical activity (median [IQR]; IPAQ)	1204 (493, 4188)	1401 (991, 2766)
Employment status, *n* (%)		
Employed	16 (80)	20 (100)
Unemployed	3 (15)	(0)
Homemaker	1 (5)	(0)
Retired	0 (0)	(0)
Occupational physical loading (OSPAQ)		
Current sitting score	770.95 (59.12)	23.64 (30.65)
Current physical labour score	31.19 (63.36)	17.14 (24.31)
Past sitting score	105.81 (136.00)	47.76 (83.53)
Past physical labour score	71.49 (87.00)	5.53 (12.66)
Smoking status, *n* (%)		
Current	1 (5)	1 (5)
Former	3 (15)	1 (5)
Never smoked	16 (80)	18 (90)

*Note:* Data are mean (standard deviation [SD]), Median (Interquartile range [IQR]) or count (percentage within‐group).

Abbreviations: IPAQ, international physical activity questionnaire–short form; OSPAQ, The occupational sitting and physical activity questionnaire for current and past work hours, multiplied by years employed.

### 3.3 Main Results

#### 3.3.1 Primary Linear Regression

Primary linear regression models are reported in Table 3. Across the total sample, BPAQ scores were negatively associated with the nucleus‐to‐annulus ratio, as assessed using average spine values from T11/T12 to L5/S1. At individual spinal levels, BPAQ scores were negatively associated with IVD T2, nucleus‐to‐annulus ratio, and positively associated with Pfirrmann grade (i.e., worse degeneration) at L5/S1, as well as negatively associated with IVD T2 at L4/L5.

**TABLE 3:** Primary linear regression models of BPAQ score and magnetic resonance IVD outcomes across all participants and stratified by sex.

All participants											
	*N*	IVD T2 (ms)		IVD height‐to‐vertebral body ratio		IVD volume (cm^3^)		Nucleus‐to‐annulus signal intensity ratio		Pfirrmann grade	
		*β* (CI)	*p*	*β* (CI)	*p*	*β* (CI)	*p*	*β* (CI)	*p*	*β* (CI)	*p*
T11/T12	40	−0.02 (−0.26, 0.23)	0.884	−0.0001 (−0.0008, 0.0006)	0.731	0.00 (−0.03, 0.03)	0.991	−0.01 (−0.02, 0.00)	0.163	0.00 (−0.01, 0.01)	0.950
T12/L1	40	−0.10 (−0.31, 0.11)	0.338	−0.0002 (−0.0008, 0.0004)	0.582	0.01 (−0.03, 0.04)	0.744	0.00 (−0.01, 0.01)	0.806	−0.01 (−0.02, 0.00)	0.228
L1/L2	40	−0.09 (−0.30, 0.13)	0.413	−0.0002 (−0.0010, 0.0006)	0.588	0.01 (−0.04, 0.06)	0.692	0.00 (−0.01, 0.01)	0.726	−0.01 (−0.01, 0.00)	0.178
L2/L3	40	−0.05 (−0.28, 0.18)	0.657	−0.0003 (−0.0010, 0.0005)	0.485	0.00 (−0.06, 0.05)	0.926	0.00 (−0.01, 0.01)	0.643	0.00 (−0.01, 0.00)	0.400
L3/L4	40	0.01 (−0.22, 0.24)	0.956	0.0001 (−0.0009, 0.0011)	0.818	0.02 (−0.04, 0.08)	0.552	0.00 (−0.01, 0.01)	0.880	0.00 (−0.02, 0.01)	0.396
L4/L5	40	−0.24 (−0.47, −0.01)	**0.041**	−0.0005 (−0.0016, 0.0005)	0.310	0.02 (−0.03, 0.06)	0.531	−0.01 (−0.03, 0.00)	0.086	0.01 (0.00, 0.02)	0.223
L5/S1	40	−0.34 (−0.61, −0.07)	**0.014**	−0.0009 (−0.0021, 0.0004)	0.171	0.02 (−0.02, 0.07)	0.349	−0.02 (−0.03, −0.01)	**0.007**	0.02 (0.01, 0.03)	**0.005**
Average spine	40	−0.07 (−0.27, 0.12)	0.448	−0.0003 (−0.0008, 0.0002)	0.269	0.01 (−0.03, 0.05)	0.682	−0.01 (−0.01, 0.00)	**0.019**	0.00 (0.00, 0.01)	0.095

Stratified by sex across all IVDs pooled											
	*N* (IVDS)	*β* (CI)	*p*	*β* (CI)	*p*	*β* (CI)	*p*	*β* (CI)	*p*	*β* (CI)	*p*
Male	140	−0.12 (−0.25, 0.01)	0.062	−0.0006 (−0.0011, 0.0000)	**0.043**	−0.01 (−0.04, 0.02)	0.534	−0.01 (−0.01, 0.00)	**0.007**	0.01 (0.00, 0.01)	**0.047**
Female	140	−0.07 (−0.19, 0.05)	0.225	0.0002 (−0.0006, 0.0009)	0.643	0.01 (−0.02, 0.05)	0.418	0.00 (−0.01, 0.01)	0.618	−0.01 (−0.02, 0.00)	**0.025**

*Note:* Data are beta coefficient (*β*) and variance as 95% confidence interval (95% CI) of the independent variable total BPAQ score and the dependent variable magnetic resonance IVD outcomes. Pfirrmann grade is reverse scale where negative values indicate less degeneration. Bold indicates a statistically significant association. Average spine is an average from spinal levels T11/T12 to L5/S1 for each participant.

Abbreviations: BPAQ, bone‐specific physical activity questionnaire; IVD, intervertebral disc; T2, time constant of the rate of proton relaxation in response to magnetisation with higher values indicating greater hydration.

Associations between BPAQ scores and individual IVDs across spinal levels T11/T12 to L5/S1 were examined separately by sex. In males, BPAQ scores were negatively associated with IVD height‐to‐vertebral body ratio and nucleus‐to‐annulus ratio and positively associated with Pfirrmann grade (i.e., worse degeneration). In females, BPAQ scores were negatively associated with Pfirrmann grade only (i.e., less degeneration).

Associations with IVD T2 were examined across the five IVD regions, from anterior to posterior, at individual spinal levels and are reported in Table 4. BPAQ scores were negatively associated with IVD T2 in the anterior annulus at L4/L5 and L5/S1, and in the anterior nucleus, central nucleus, and posterior nucleus at L5/S1, but not in the posterior annulus.

**TABLE 4:** Primary linear regression models of BPAQ scores associations with magnetic resonance IVD T2 across five anterior to posterior regions.

IVD T2 (ms)	*n*	Region 1		Region 2		Region 3		Region 4		Region 5	
		*β* (95% CI)	*p*	*β* (95% CI)	*p*	*β* (95% CI)	*p*	*β* (95% CI)	*p*	*β* (95% CI)	*p*
T11/T12	40	−0.02 (−0.21, 0.17)	0.833	−0.08 (−0.41, 0.25)	0.619	−0.07 (−0.48, 0.34)	0.733	0.10 (−0.36, 0.56)	0.666	0.25 (−0.01, 0.51)	0.059
T12/L1	40	−0.09 (−0.27, 0.09)	0.318	0.01 (−0.32, 0.34)	0.964	−0.06 (−0.42, 0.30)	0.733	−0.03 (−0.51, 0.44)	0.894	0.04 (−0.35, 0.43)	0.844
L1/L2	40	−0.02 (−0.24, 0.20)	0.848	0.00 (−0.34, 0.35)	0.990	0.01 (−0.40, 0.42)	0.967	−0.05 (−0.56, 0.45)	0.835	−0.05 (−0.37, 0.26)	0.741
L2/L3	40	−0.03 (−0.24, 0.18)	0.754	0.03 (−0.36, 0.41)	0.895	0.06 (−0.37, 0.50)	0.774	−0.13 (−0.57, 0.31)	0.539	−0.09 (−0.35, 0.17)	0.470
L3/L4	40	−0.03 (−0.30, 0.23)	0.794	0.11 (−0.33, 0.56)	0.602	0.12 (−0.39, 0.64)	0.629	0.01 (−0.50, 0.52)	0.972	−0.17 (−0.36, 0.01)	0.064
L4/L5	40	−0.21 (−0.37, −0.04)	**0.017**	−0.41 (−0.91, 0.08)	0.100	−0.45 (−1.00, 0.11)	0.110	−0.39 (−0.81, 0.03)	0.067	−0.09 (−0.24, 0.06)	0.234
L5/S1	40	−0.40 (−0.65, −0.14)	**0.003**	−0.63 (−1.12, −0.14)	**0.013**	−0.62 (−1.15, −0.09)	**0.022**	−0.51 (−0.91, −0.12)	**0.012**	−0.15 (−0.42, 0.11)	0.252

*Note:* Data are beta coefficient (*β*) and variance as 95% confidence interval (95% CI) of the independent variable total BPAQ score and the dependent variable magnetic resonance IVD T2 values in five anterior to posterior IVD regions. Bold indicates a statistically significant association.

Abbreviations: BPAQ, bone‐specific physical activity questionnaire; IVD, intervertebral disc; region 1, anterior annulus; region 2, posterior nucleus; region 3, central nucleus; region 4, posterior nucleus; region 5, posterior annulus; T2, time constant of the rate of proton relaxation in response to magnetisation with higher values indicating greater hydration.

#### 3.3.2 Multiple Linear Regression

**FIGURE 3:**
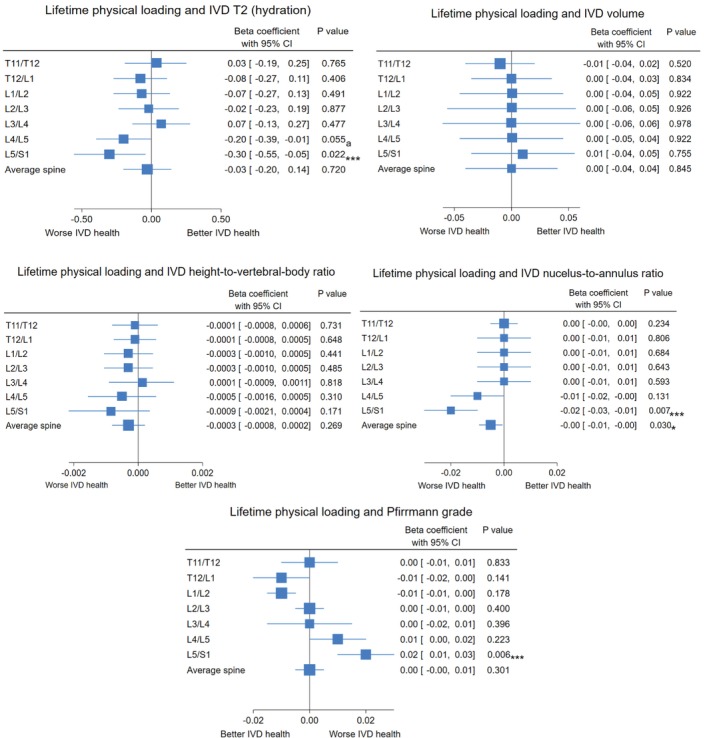
Multiple linear regression (final model) showing the association between BPAQ score and magnetic resonance IVD outcomes with statistically significant covariates controlled for across all participants at individual spinal levels and the average spine. Data are Beta coefficient (*β*) and variance as 95% confidence interval (95% CI) of the independent variable total BPAQ score and the dependent variable magnetic resonance IVD outcomes. Significant covariates controlled for included age, body mass index, current occupational sitting, current occupational heavy labour, past occupational sitting, and past occupational heavy labour. Average spine is an average from spinal levels T11/T12 to L5/S1 for each participant. Pfirrmann grade is a reverse scale where negative values indicate less degeneration. *Indicates a statistically significant association; a: Indicates univariate regression significance lost when controlling for significant covariates; ***Indicates significance after adjusting for type I error. BPAQ: Bone‐specific Physical Activity Questionnaire; IVD: Intervertebral disc; T2: Time constant of the rate of proton relaxation in response to magnetisation, with higher values indicating greater hydration.

All covariate linear regression models are reported in Supporting Information B and final multiple linear regression models with adjustment for significant covariates are shown in Figure 3. The nucleus‐to‐annulus signal intensity ratio remained negatively associated with BPAQ scores when examined using average spine values.

At L5/S1, BPAQ scores remained negatively associated with IVD T2 and nucleus‐to‐annulus signal intensity ratio and positively associated with Pfirrmann grade (i.e., greater degeneration). No other individual spinal levels revealed significant associations with BPAQ scores after covariate adjustment.

**FIGURE 4:**
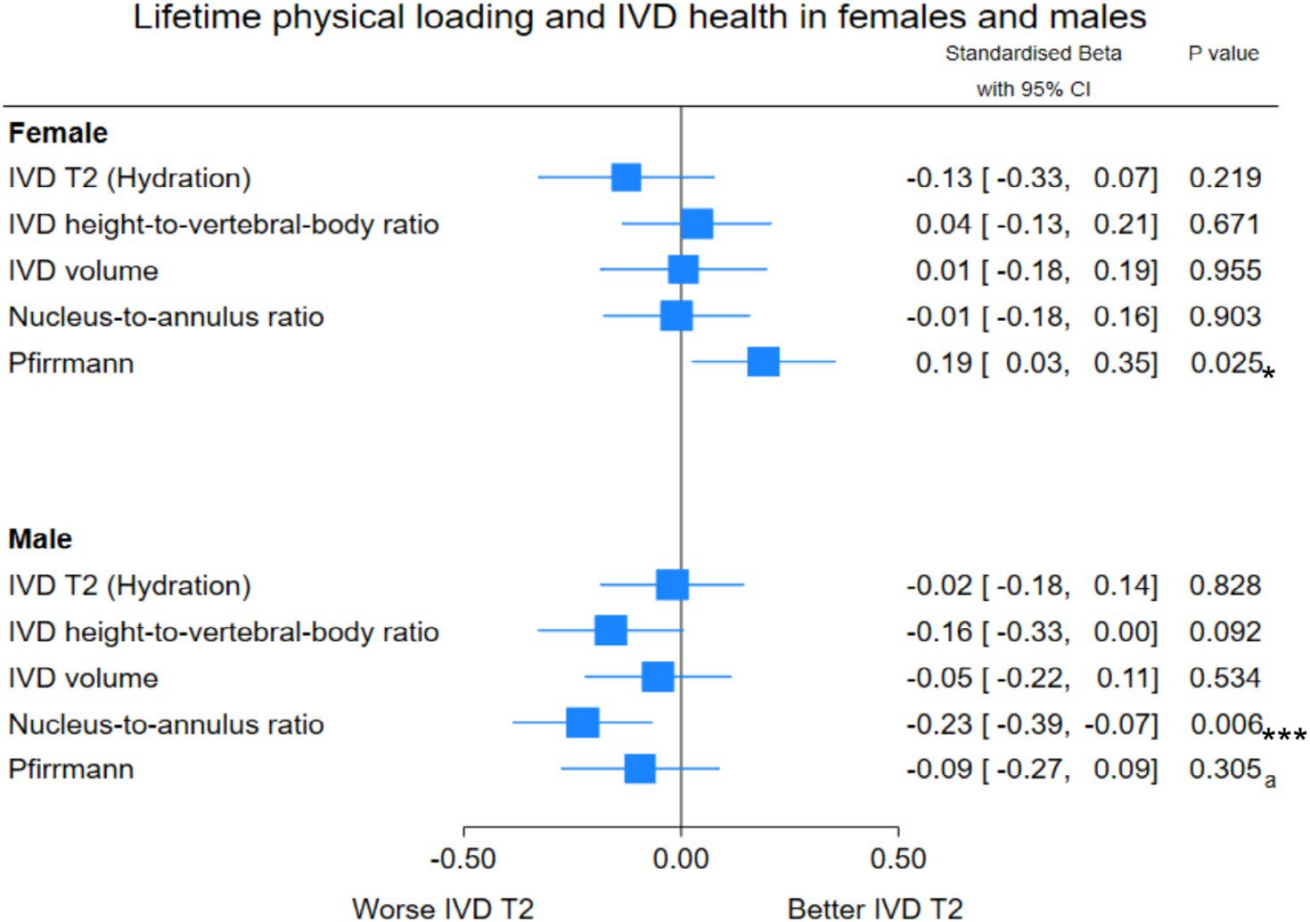
Multiple linear regression (final model) showing BPAQ score associations with magnetic resonance IVD outcomes with statistically significant covariates controlled for across all IVDs pooled from T11/T12 to L5/S1 stratified by sex. Data are standardised Beta coefficient (β*) and variance as 95% confidence interval (95% CI) of the independent variable total BPAQ score and the dependent variable magnetic resonance IVD outcomes. Significant covariates controlled for included age, body mass index, current occupational sitting, current occupational heavy labour, past occupational sitting, and past occupational heavy labour. Pfirrmann grade coefficients are presented as inverse values to align with the direction of other outcomes (higher values indicate better IVD health). *Indicates a statistically significant association; a: Indicates univariate regression significance lost when controlling for significant covariates; ***Indicates significance after adjusting for type I error. BPAQ: Bone‐specific Physical Activity Questionnaire; IVD: Intervertebral disc; T2: Time constant of the rate of proton relaxation in response to magnetisation with higher values indicating greater hydration.

When all IVDs were stratified by sex, male BPAQ scores were negatively associated with nucleus‐to‐annulus signal intensity ratio and female BPAQ scores were negatively associated with Pfirrmann grade (i.e., less degeneration; Figure 4).

**FIGURE 5:**
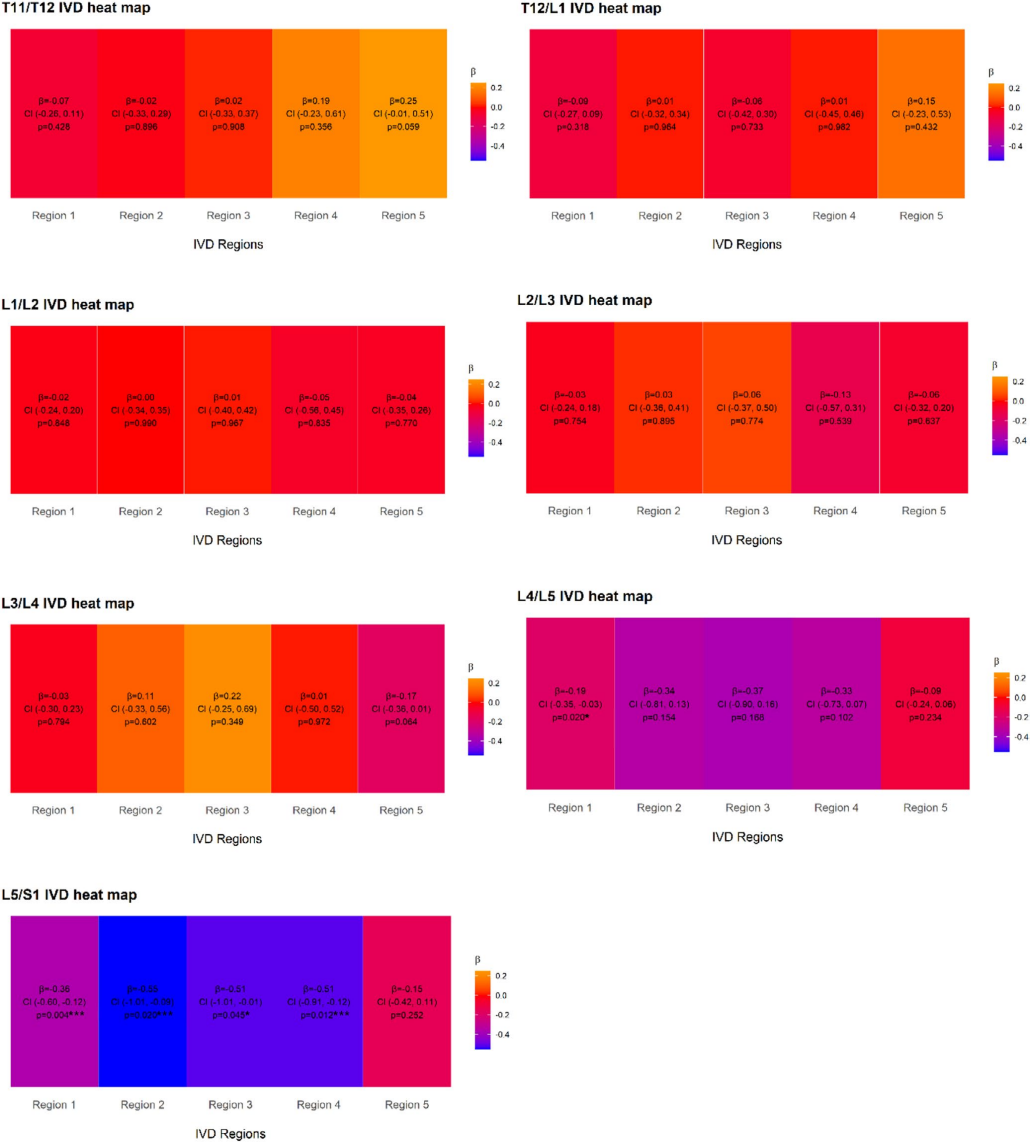
Final multiple linear regression model: Heat maps showing BPAQ score associations with T2 values in five IVD regions with statistically significant covariates controlled for at individual spinal levels. Data are Beta coefficient (*β*) and variance as 95% confidence interval (95% CI) of the independent variable total BPAQ score and the dependent variable magnetic resonance IVD T2 values in five IVD regions. Significant covariates controlled for included age, body mass index, current occupational sitting, current occupational heavy labour, past occupational sitting, and past occupational heavy labour. *Indicates a statistically significant association; ***Indicates significance after adjusting for type I error. BPAQ: Bone‐specific Physical Activity Questionnaire; IVD: Intervertebral disc; region 1: Anterior annulus, region 2: Posterior nucleus, region 3: Central nucleus, region 4: Posterior nucleus, region 5: Posterior annulus; T2: Time constant of the rate of proton relaxation in response to magnetisation with higher values indicating greater hydration.

Across the five IVD regions, the multiple linear regression models revealed that IVD T2 remained negatively associated with BPAQ scores in the anterior annulus at L4/L5 and L5/S1, as well as in all other regions except the posterior annulus at L5/S1 (Figure 5).

No pain or disability outcomes were associated with BPAQ scores (Table 5).

**TABLE 5:** Primary linear regression models of total BPAQ scores and participant‐reported pain and disability.

	*n*	*β* (95% CI)	*p*
Average pain (VAS)	40	−0.03 (−0.12, 0.06)	0.559
Current pain (VAS)	40	−0.06 (−0.15, 0.02)	0.144
Worst pain (VAS)	40	0.03 (−0.05, 0.12)	0.448
Disability (ODI)	40	0.20 (−0.01, 0.41)	0.059

*Note:* Data are Beta coefficient (*β*) and variance as 95% confidence interval (95% CI) of the independent variable total BPAQ score and the dependent variable pain and disability outcomes.

Abbreviations: VAS, visual analogue scale; ODI, Oswestry disability index.

### 3.4 Sensitivity Analyses

After the false discovery rate correction, multiple linear regression models remained significant at L5/S1 for IVD T2, nucleus‐to‐annulus ratio, and Pfirrmann grade. When stratified by sex, the nucleus‐to‐annulus ratio remained significant across male IVDs. Across the five IVD regions, L5/S1 IVD T2 remained significant in the anterior annulus, anterior nucleus, and posterior nucleus.

### 3.5 Harms

There were no participant adverse events.

## 4 Discussion

The current study examined the relationship between lifetime physical loading as measured by the BPAQ and magnetic resonance IVD measures, while controlling for several covariates in a population likely to have IVD degeneration. After adjusting for covariates and risk of type I errors, results showed that in adults with chronic low back pain, indicators of worse IVD health were associated with greater lifetime physical loading at the L5/S1 spinal level. Specifically, as measured by the nucleus‐to‐annulus ratio, Pfirrmann grade, and whole IVD T2, as well as its regional components, the anterior annulus, anterior nucleus, and posterior nucleus. IVD outcomes at other spinal levels and across average spine values were not associated with lifetime physical loading. Sex‐stratified analysis showed that greater physical loading was associated with a lower nucleus‐to‐annulus ratio in males. Lastly, BPAQ scores were not associated with pain and disability outcomes.

Our observations in participant‐level average spine data indicate that BPAQ scores were not associated with IVD health. These findings suggest that optimal loading for IVDs may differ from patterns reported for bone mineral density [26]. Bone remodelling is driven by high internal forces and mechanical strain, primarily from muscle contractions [47]. Thus, activities that produce high‐magnitude ground reaction forces, performed with sufficient frequency and volume, are commonly associated with osteogenic stimulus [47]. The ideal physical loading parameters to stimulate IVD ‘remodelling’ are less clear; however, we speculate based on these findings that the lifetime physical activity loading required for IVD health likely differs from that required for bone health. Limited research exploring IVD loading suggests that a high magnitude of physical loading may be associated with poor IVD health, as seen in cross‐sectional studies of elite athletes [14, 15, 16] and supported by some of our results. The BPAQ may still have utility in quantifying IVD health, which likely has a non‐linear relationship. Some physical loading appears vital for maintaining IVD homeostasis and promoting IVD health [48], with evidence suggesting moderate physical loading is the most beneficial [49]. Furthermore, greater evidence suggests that no physical loading, as seen in sedentary populations [12] or extended bed rest [50], is detrimental to IVD health. Our observed lack of confident findings in either direction (for most outcomes) aligns with these suggestions, given that higher and lower lifetime physical loadings may have mutually negated each other out and led to a lack of significant findings. Understanding the appropriate physical loading for IVD adaptations via prospective studies will help guide the development of a novel questionnaire designed to predict IVD health based on exposure to physical loading.

Poorer IVD health was associated with higher lifetime physical loading scores at L5/S1. These findings were consistent across the three IVD outcomes likely to be the most sensitive to early changes in IVD health (T2, nucleus‐to‐annulus ratio, and Pfirrmann grade), opposed to IVD height‐to‐vertebral ratio and volume, which likely change secondary to the aforementioned outcomes. L5/S1 is the most common spinal level susceptible to degeneration [51] due to the higher weight‐bearing loads, including the transfer of spinal loads onto the sacrum and lower limbs [52]. This may explain why higher lifetime ground reaction forces measured by BPAQ were associated with poorer IVD health at this level only. Across the literature, cross‐sectional studies consistently show sensitivity to physical loading exposures at L5/S1, both positively (running [18, 19] and physical activity [53]) and negatively (rowing [54] and weightlifting) [14], albeit also in other lumbar levels, with only one study in long‐distance runners revealing differences at L5/S1 only [19]. While observational, the positive and negative associations suggest the type of physical loading may determine whether the IVD adapts positively or negatively, whereas the BPAQ tool assumes that more ground reaction force is better. These observations suggest that the BPAQ may detect poorer IVD health at L5/S1 but not at other spinal levels.

Interestingly, the only positive association between physical loading and better IVD health appeared in females, based on Pfirrmann grade. This association did not persist after controlling for Type I errors; however, its opposite direction relative to all other findings merits further consideration. These data suggest that greater lifetime exposure to physical loading was associated with less IVD degeneration, whereas this was not evident in males. Further, the negative association between BPAQ and the nucleus‐to‐annulus ratio in males was not observed in females. One possible explanation may relate to current habitual physical activity levels. Females reported higher median IPAQ scores with less variability (narrower interquartile range) than males. This pattern may indicate a more consistent and preferred volume of physical loading among females, with fewer extremes across the sample, which could have contributed to statistically significant results. The different observations in males and females are supported by recent literature, which suggests that tissue adaptation in response to physical loading is influenced by sex‐specific factors, including muscle [55] and spinal tissue [56]. A recent randomised controlled trial showed that a strength and conditioning intervention reduced vertebral bone marrow adipose tissue in males, yet not in females [56]. Sex differences may be influenced by molecular, hormone‐dependent, and epigenomic factors, which could be accounted for in research moving forward (e.g., by including both sexes, menstrual cycle tracking, and conducting large and ethnically diverse studies) [55]. Our observational findings, whilst limited in drawing causal conclusions and with potential risk for type I errors, support including biological sex‐stratified analyses in future attempts to understand how exposure to physical loading influences spinal physiology.

### 4.1 Clinical Implications

The current study suggests that physical loading may be a modifiable factor in IVD health, albeit in a direction opposite to that observed for bone health. Further, lifetime physical loading was not associated with pain and disability in individuals with chronic low back pain; thus, physical loading alone does not appear helpful in determining clinical status in this population. The BPAQ tool may be appropriate to determine poor IVD health at L5/S1 in individuals experiencing chronic low back pain. Future studies could compare a larger cohort of individuals with and without chronic low back pain to determine whether these observations are specific to this clinical population. Moreover, a more IVD‐specific tool that aligns with positive IVD adaptations likely needs to assign negative values to high sedentary time and to physical loading that exceeds a threshold for ground reaction forces. However, the exact point at which this threshold should be set remains unclear. Lastly, when considering exercise prescription to support both bone and IVD health, a multimodal exercise approach may be beneficial, as different exercise modalities may influence each musculoskeletal tissue in distinct ways.

### 4.2 Strengths and Limitations

Our study was strengthened by using gold‐standard magnetic resonance imaging for IVD measures and controlling for significant covariates known to influence early and more progressed IVD degeneration. However, our study was not without limitations. First, reliance on subjective recall of lifetime physical loading using the BPAQ tool is problematic, as recall spans the whole lifetime, and early years may not be accurately recalled. Second, the generalisability of our observations is limited to individuals with chronic low back pain, specifically those with a pre‐existing interest in exercise training (due to recruitment for the larger randomised controlled trial). Therefore, our findings are not generalisable to populations who are pain‐free or disinterested in exercise. Third, analyses using average spine values may be subject to within‐participant correlation that was not explicitly modelled; however, this was addressed through level‐specific analyses. Fourth, the Occupational Sitting and Physical Activity Questionnaire showed weak correlations for assessing occupational heavy labour, limiting confidence in its assessment of the confounding effect.

## 5 Conclusion

Higher lifetime physical loading, as measured by the BPAQ, was associated with poorer IVD health at the L5‐S1 spinal level. We suggest that the lifetime physical activity loading required for IVD health differs from that observed with bone when assessed using the BPAQ. A more appropriate tool would need to consider which loading conditions are beneficial for the IVD, for example, assigning positive values to moderate physical loading and negative values to both sedentary and high‐loading activities.

## Author Contributions

**S.D.T., D.L.B., U.H.M., P.J.O.:** conceptualization. **C.L.S., C.N., M.C., A.I., U.H.M, H.R.N.:** data curation. **C.L.S., P.J.O.:** formal analysis, software, visualization. **D.L.B., U.H.M., DS, PJO.:** funding acquisition. **C.L.S., C.N., P.J.O.:** investigation. **C.L.S., C.N., S.D.T., P.J.O.:** project administration. **P.J.O.:** resources. **C.T.M., N.L.M., D.S., P.J.O., P.B.:** supervision. **P.J.O., S.D.T.:** validation. **C.L.S.:** writing – original draft. All: methodology, writing – review and editing.

## Funding

This work was supported by internal funding (Deakin University). S.D.T. is supported by a University of Melbourne Sir Randal Heymanson Fellowship. D.S. is supported by a National Health and Medical Research Council Investigator Grant (GNT1174886). C.L.S. and C.N. are supported by Australian Government Research Training Program (RTP) Scholarships.

## Ethics Statement

Ethics approval was provided by the Deakin University Human Research Ethics Committee (ID: 2022‐162) on 26 September 2022.

## Conflicts of Interest

The authors declare no conflicts of interest.

## Supporting information


**Supporting Information: A** STROBE Statement—checklist of items that should be included in reports of observational studies.
**Supporting Information: B:** Linear regression models of potential covariates.
**Table S1:** Univariate linear regression of potential covariates with magnetic resonance IVD outcomes across all participants.
**Table S2:** Univariate linear regression of potential covariates with magnetic resonance IVD outcomes across male participants.
**Table S3:** Univariate linear regression of potential covariates with magnetic resonance IVD outcomes across female participants.
**Table S4:** Univariate linear regression of potential covariates with magnetic resonance IVD T2 across five IVD regions.

## Data Availability

The data that support the findings of this study are available from the corresponding author upon reasonable request.
