# Supplementary material for: Lifetime Physical Loading and Magnetic Resonance‐Derived Intervertebral Disc Health in Adults With Chronic Low Back Pain: A Cross‐Sectional Study
Source: JOR Spine. 2026 May 15;9(2):e70186. doi: 10.1002/jsp2.70186 (PMC13179145; doi:10.1002/jsp2.70186)
Supplement: Supplementary file 1 — Supporting Information: A STROBE Statement—checklist of items that should be included in reports of observational studies. Supporting Information: B: Linear regression models of potential covariates. Table S1: Univariate linear regression of potential covariates with magnetic resonance IVD outcomes across all participants. Table S2: Univariate linear regression of potential covariates with magnetic resonance IVD outcomes across male participants. Table S3: Univariate linear regression of potential covariates with magnetic resonance IVD outcomes across female participants. Table S4: Univariate linear regression of potential covariates with magnetic resonance IVD T2 across five IVD regions. [file JSP2-9-e70186-s001.docx]

**SUPPLEMENT**

Contents

[Supplement A: STROBE Statement—checklist of items that should be included in reports of observational studies. 2](#_Toc227830242)

[Supplement B: Linear regression models of potential covariates 5](#_Toc227830243)

# Supplement A: STROBE Statement—checklist of items that should be included in reports of observational studies.

STROBE Statement—Checklist of items that should be included in reports of ***cross-sectional studies***

|  | Item No | Recommendation | Reported |
| --- | --- | --- | --- |
| **Title and abstract** | 1 | (*a*) Indicate the study’s design with a commonly used term in the title or the abstract | Yes |
|  |  | (*b*) Provide in the abstract an informative and balanced summary of what was done and what was found | Yes |
| Introduction | | |  |
| Background/rationale | 2 | Explain the scientific background and rationale for the investigation being reported | Yes |
| Objectives | 3 | State specific objectives, including any prespecified hypotheses | Yes |
| Methods | | |  |
| Study design | 4 | Present key elements of study design early in the paper | Yes |
| Setting | 5 | Describe the setting, locations, and relevant dates, including periods of recruitment, exposure, follow-up, and data collection | Yes |
| Participants | 6 | (*a*) Give the eligibility criteria, and the sources and methods of selection of participants | Yes |
| Variables | 7 | Clearly define all outcomes, exposures, predictors, potential confounders, and effect modifiers. Give diagnostic criteria, if applicable | Yes |
| Data sources/ measurement | 8* | For each variable of interest, give sources of data and details of methods of assessment (measurement). Describe comparability of assessment methods if there is more than one group | Yes |
| Bias | 9 | Describe any efforts to address potential sources of bias | Yes |
| Study size | 10 | Explain how the study size was arrived at | Yes |
| Quantitative variables | 11 | Explain how quantitative variables were handled in the analyses. If applicable, describe which groupings were chosen and why | Yes |
| Statistical methods | 12 | (*a*) Describe all statistical methods, including those used to control for confounding | Yes |
|  |  | (*b*) Describe any methods used to examine subgroups and interactions | Yes |
|  |  | (*c*) Explain how missing data were addressed | Yes |
|  |  | (*d*) If applicable, describe analytical methods taking account of sampling strategy | Yes |
|  |  | (*e*) Describe any sensitivity analyses | Yes |
| Results | | |  |
| Participants | 13* | (a) Report numbers of individuals at each stage of study—eg numbers potentially eligible, examined for eligibility, confirmed eligible, included in the study, completing follow-up, and analysed | Yes |
|  |  | (b) Give reasons for non-participation at each stage | Yes |
|  |  | (c) Consider use of a flow diagram | Yes |
| Descriptive data | 14* | (a) Give characteristics of study participants (eg demographic, clinical, social) and information on exposures and potential confounders | Yes |
|  |  | (b) Indicate number of participants with missing data for each variable of interest | Yes |
| Outcome data | 15* | Report numbers of outcome events or summary measures | Yes |
| Main results | 16 | (*a*) Give unadjusted estimates and, if applicable, confounder-adjusted estimates and their precision (eg, 95% confidence interval). Make clear which confounders were adjusted for and why they were included | Yes |
|  |  | (*b*) Report category boundaries when continuous variables were categorized | Yes |
|  |  | (*c*) If relevant, consider translating estimates of relative risk into absolute risk for a meaningful time period | Yes |
| Other analyses | 17 | Report other analyses done—eg analyses of subgroups and interactions, and sensitivity analyses | Yes |
| Discussion | | |  |
| Key results | 18 | Summarise key results with reference to study objectives | Yes |
| Limitations | 19 | Discuss limitations of the study, taking into account sources of potential bias or imprecision. Discuss both direction and magnitude of any potential bias | Yes |
| Interpretation | 20 | Give a cautious overall interpretation of results considering objectives, limitations, multiplicity of analyses, results from similar studies, and other relevant evidence | Yes |
| Generalisability | 21 | Discuss the generalisability (external validity) of the study results | Yes |
| Other information | | |  |
| Funding | 22 | Give the source of funding and the role of the funders for the present study and, if applicable, for the original study on which the present article is based | Yes |
| Quantitative variables | 11 | Explain how quantitative variables were handled in the analyses. If applicable, describe which groupings were chosen and why | Yes |
| Statistical methods | 12 | (a) Describe all statistical methods, including those used to control for confounding | Yes |
|  |  | (b) Describe any methods used to examine subgroups and interactions | Yes |
|  |  | (c) Explain how missing data were addressed | Yes |
|  |  | (d) Cohort study—If applicable, explain how loss to follow-up was addressed  Case-control study—If applicable, explain how matching of cases and controls was addressed  Cross-sectional study—If applicable, describe analytical methods taking account of sampling strategy | Yes |
|  |  | (e) Describe any sensitivity analyses | Yes |
| Participants | 13* | (a) Report numbers of individuals at each stage of study—eg numbers potentially eligible, examined for eligibility, confirmed eligible, included in the study, completing follow-up, and analysed | Yes |
|  |  | (b) Give reasons for non-participation at each stage | Yes |
|  |  | (c) Consider use of a flow diagram | Yes |
| Descriptive data | 14* | (a) Give characteristics of study participants (eg demographic, clinical, social) and information on exposures and potential confounders | Yes |
|  |  | (b) Indicate number of participants with missing data for each variable of interest | Yes |
|  |  | (c) Cohort study—Summarise follow-up time (eg, average and total amount) | Yes |
| Outcome data | 15* | Cohort study—Report numbers of outcome events or summary measures over time | Yes |
|  |  | Case-control study—Report numbers in each exposure category, or summary measures of exposure | Yes |
|  |  | Cross-sectional study—Report numbers of outcome events or summary measures | Yes |
| Main results | 16 | (a) Give unadjusted estimates and, if applicable, confounder-adjusted estimates and their precision (eg, 95% confidence interval). Make clear which confounders were adjusted for and why they were included | Yes |
|  |  | (b) Report category boundaries when continuous variables were categorized | Yes |
|  |  | (c) If relevant, consider translating estimates of relative risk into absolute risk for a meaningful time period | Yes |

*Give information separately for exposed and unexposed groups.

**Note:** An Explanation and Elaboration article discusses each checklist item and gives methodological background and published examples of transparent reporting. The STROBE checklist is best used in conjunction with this article (freely available on the Web sites of PLoS Medicine at http://www.plosmedicine.org/, Annals of Internal Medicine at http://www.annals.org/, and Epidemiology at http://www.epidem.com/). Information on the STROBE Initiative is available at www.strobe-statement.org.

| Other analyses | 17 | Report other analyses done—eg analyses of subgroups and interactions, and sensitivity analyses | Yes |
| --- | --- | --- | --- |
| Discussion | | | |
| Key results | 18 | Summarise key results with reference to study objectives | Yes |
| Limitations | 19 | Discuss limitations of the study, taking into account sources of potential bias or imprecision. Discuss both direction and magnitude of any potential bias | Yes |
| Interpretation | 20 | Give a cautious overall interpretation of results considering objectives, limitations, multiplicity of analyses, results from similar studies, and other relevant evidence | Yes |
| Generalisability | 21 | Discuss the generalisability (external validity) of the study results | Yes |
| Other information | |  | |
| Funding | 22 | Give the source of funding and the role of the funders for the present study and, if applicable, for the original study on which the present article is based | Yes |

*Give information separately for cases and controls in case-control studies and, if applicable, for exposed and unexposed groups in cohort and cross-sectional studies

# Supplement B: Linear regression models of potential covariates

**Table A.** Univariate linear regression of potential covariates with magnetic resonance IVD outcomes across all participants.

| **Potential covariate outcomes** | | | | | | | | | | | | | | | | | | | | | | | | | |
| --- | --- | --- | --- | --- | --- | --- | --- | --- | --- | --- | --- | --- | --- | --- | --- | --- | --- | --- | --- | --- | --- | --- | --- | --- | --- |
|  | | **Age** | | **BMI** | | | | **Current occupational sitting** | | | | | **Current occupational labour** | | | | **Past occupational sitting** | | | **Past occupational labour** | | | | | |
|  | **n** | **β (CI)** | **P value** | **β (CI)** | | **P value** | **β (CI)** | | | **P value** | **β (CI)** | | | | **P value** | **β (CI)** | | | **P value** | **β (CI)** | | | | **P value** | |
| **IVD T2 (ms)** | | | | | | | | | | | | | | | | | | | | | | | | | |
| **T11/T12** | 40 | -0.82 (-1.39, -0.24) | **0.007** | -0.65 (-1.16, -0.14) | | **0.013** | -0.07 (-0.15, 0.00) | | | 0.060 | -0.02 (-0.11, 0.07) | | | | 0.698 | -0.02 (-0.05, 0.02) | | | 0.410 | -0.01 (-0.07, 0.06) | | | | 0.825 | |
| **T12/L1** | 40 | -0.51 (-1.04, 0.02) | 0.057 | -0.61 (-1.04, -0.18) | | **0.007** | -0.04 (-0.11, 0.03) | | | 0.231 | -0.03 (-0.10, 0.05) | | | | 0.432 | 0.00 (-0.03, 0.03) | | | 0.888 | -0.02 (-0.07, 0.04) | | | | 0.522 | |
| **L1/L2** | 40 | -0.34 (-0.89, 0.21) | 0.214 | -0.54 (-0.98, -0.09) | | **0.021** | -0.04 (-0.10, 0.03) | | | 0.243 | -0.01 (-0.08, 0.07) | | | | 0.815 | -0.01 (-0.04, 0.02) | | | 0.448 | -0.02 (-0.07, 0.03) | | | | 0.361 | |
| **L2/L3** | 40 | -0.67 (-1.22, -0.12) | **0.018** | -0.48 (-0.96, 0.01) | | 0.054 | -0.06 (-0.13, 0.01) | | | 0.080 | 0.01 (-0.07, 0.09) | | | | 0.771 | -0.01 (-0.04, 0.02) | | | 0.588 | -0.02 (-0.08, 0.03) | | | | 0.421 | |
| **L3/L4** | 40 | -0.82 (-1.35, -0.28) | **0.004** | -0.42 (-0.92, 0.08) | | 0.095 | -0.10 (-0.17, -0.03) | | | **0.008** | -0.02 (-0.11, 0.06) | | | | 0.627 | -0.02 (-0.05, 0.02) | | | 0.362 | -0.01 (-0.07, 0.05) | | | | 0.822 | |
| **L4/L5** | 40 | -0.98 (-1.53, -0.43) | **0.001** | -0.55 (-1.07, -0.03) | | **0.039** | -0.03 (-0.11, 0.04) | | | 0.387 | 0.00 (-0.08, 0.08) | | | | 0.988 | -0.02 (-0.05, 0.01) | | | 0.193 | -0.02 (-0.07, 0.04) | | | | 0.524 | |
| **L5/S1** | 40 | -0.89 (-1.59, -0.19) | **0.014** | -0.44 (-1.08, 0.20) | | 0.170 | -0.08 (-0.18, 0.01) | | | 0.082 | -0.05 (-0.16, 0.06) | | | | 0.349 | -0.01 (-0.05, 0.03) | | | 0.643 | -0.04 (-0.11, 0.03) | | | | 0.285 | |
| **Average spine (T11/T12-L5/S1)** | 40 | -0.76 (-1.20, -0.31) | **0.002** | -0.49 (-0.90, -0.08) | | **0.021** | -0.05 (-0.11, 0.02) | | | 0.151 | -0.00 (-0.08, 0.07) | | | | 0.896 | -0.01 (-0.04, 0.01) | | | 0.328 | -0.02 (-0.07, 0.03) | | | | 0.366 | |
| **All IVDs** | 280 | -0.72 (-0.93, -0.50) | **<0.001** | -0.53 (-0.71, -0.34) | | **<0.001** | -0.06 (-0.09, -0.03) | | | **<0.001** | -0.02 (-0.05, 0.02) | | | | 0.308 | -0.01 (-0.02, 0.00) | | | 0.079 | -0.02 (-0.04, 0.00) | | | | 0.087 | |
| **IVD height-to-vertebral body ratio** | | | | |  | | |  | |  | |  | |  | | | |  | |  | |  | | | |
| **T11/T12** | 40 | -0.00 (-0.00, 0.00) | 0.530 | 0.00 (-0.00, 0.00) | | 0.620 | 0.00 (-0.00, 0.00) | | | 0.200 | 0.00 (-0.00, 0.00) | | | | 0.990 | 0.00 (-0.00, 0.00) | | | 0.130 | 0.00 (-0.00, 0.00) | | | | 0.776 | |
| **T12/L1** | 40 | -0.00 (-0.00, 0.00) | 0.928 | 0.00 (-0.00, 0.00) | | 0.190 | 0.00 (-0.00, 0.00) | | | 0.133 | 0.00 (-0.00, 0.00) | | | | 0.610 | 0.00 (0.00, 0.00) | | | **0.039** | 0.00 (-0.00, 0.00) | | | | 0.671 | |
| **L1/L2** | 40 | 0.00 (-0.00, 0.00) | 0.314 | 0.00 (-0.00, 0.00) | | 0.233 | 0.00 (0.00, 0.00) | | | **0.012** | 0.00 (-0.00, 0.00) | | | | 0.650 | 0.00 (-0.00, 0.00) | | | 0.279 | 0.00 (-0.00, 0.00) | | | | 0.816 | |
| **L2/L3** | 40 | 0.00 (-0.00, 0.00) | 0.319 | 0.00 (-0.00, 0.00) | | 0.373 | 0.00 (-0.00, 0.00) | | | 0.418 | 0.00 (-0.00, 0.00) | | | | 0.469 | 0.00 (-0.00, 0.00) | | | 0.377 | 0.00 (-0.00, 0.00) | | | | 0.594 | |
| **L3/L4** | 40 | 0.00 (-0.00, 0.00) | 0.609 | 0.00 (-0.00, 0.00) | | 0.262 | 0.00 (-0.00, 0.00) | | | 0.828 | 0.00 (-0.00, 0.00) | | | | 0.610 | 0.00 (-0.00, 0.00) | | | 0.415 | 0.00 (-0.00, 0.00) | | | | 0.489 | |
| **L4/L5** | 40 | 0.00 (-0.00, 0.00) | 0.307 | -0.00 (-0.00, 0.00) | | 0.878 | 0.00 (-0.00, 0.00) | | | 0.974 | 0.00 (-0.00, 0.00) | | | | 0.503 | 0.00 (0.00, 0.00) | | | 0.019 | 0.00 (-0.00, 0.00) | | | | 0.816 | |
| **L5/S1** | 40 | -0.00 (-0.00, 0.00) | 0.940 | 0.00 (-0.00, 0.00) | | 0.997 | -0.00 (-0.00, 0.00) | | | 0.171 | -0.00 (-0.00, 0.00) | | | | 0.863 | 0.00 (-0.00, 0.00) | | | 0.879 | 0.00 (-0.00, 0.00) | | | | 0.673 | |
| **Average spine (T11/T12-L5/S1)** | 40 | 0.00 (-0.00, 0.00) | 0.547 | 0.00 (-0.00, 0.00) | | 0.515 | 0.00 (-0.00, 0.00) | | | 0.194 | 0.00 (-0.00, 0.00) | | | | 0.790 | 0.00 (-0.00, 0.00) | | | 0.087 | 0.00 (-0.00, 0.00) | | | | 0.663 | |
| **All IVDs** | 280 | 0.00 (-0.00, 0.00) | 0.411 | 0.00 (-0.00, 0.00) | | 0.232 | 0.00 (-0.00, 0.00) | | | 0.443 | 0.00 (-0.00, 0.00) | | | | 0.595 | 0.00 (0.00, 0.00) | | | **0.016** | 0.00 (-0.00, 0.00) | | | | 0.781 | |
| **IVD volume (cm^3)^** | | | | | |  |  | | |  |  | | | |  |  | | |  |  | | | |  | |
| **T11/T12** | 40 | 0.06 (-0.03, 0.14) | 0.174 | 0.03 (-0.04, 0.10) | | 0.409 | 0.01 (0.00, 0.02) | | | **0.007** | 0.00 (-0.01, 0.02) | | | | 0.444 | 0.00 (-0.00, 0.01) | | | 0.078 | 0.01 (0.00, 0.02) | | | | **0.038** | |
| **T12/L1** | 40 | 0.05 (-0.05, 0.15) | 0.314 | 0.04 (-0.04, 0.13) | | 0.297 | 0.01 (-0.00, 0.02) | | | 0.066 | 0.00 (-0.01, 0.02) | | | | 0.861 | 0.00 (-0.00, 0.01) | | | 0.283 | 0.01 (0.00, 0.02) | | | | **0.019** | |
| **L1/L2** | 40 | 0.07 (-0.06, 0.21) | 0.271 | 0.04 (-0.08, 0.16) | | 0.492 | 0.02 (0.01, 0.04) | | | **0.009** | 0.01 (-0.01, 0.02) | | | | 0.534 | 0.00 (-0.00, 0.01) | | | 0.332 | 0.01 (-0.00, 0.02) | | | | 0.055 | |
| **L2/L3** | 40 | 0.10 (-0.04, 0.24) | 0.151 | 0.06 (-0.06, 0.18) | | 0.307 | 0.02 (-0.00, 0.03) | | | 0.054 | 0.00 (-0.02, 0.02) | | | | 0.765 | 0.00 (-0.00, 0.01) | | | 0.297 | 0.01 (-0.00, 0.02) | | | | 0.125 | |
| **L3/L4** | 40 | 0.11 (-0.05, 0.27) | 0.157 | 0.10 (-0.04, 0.24) | | 0.150 | 0.02 (-0.00, 0.04) | | | 0.114 | 0.01 (-0.02, 0.03) | | | | 0.599 | 0.00 (-0.01, 0.01) | | | 0.355 | 0.02 (0.01, 0.03) | | | | **0.009** | |
| **L4/L5** | 40 | 0.13 (0.01, 0.25) | **0.038** | 0.05 (-0.06, 0.16) | | 0.341 | 0.01 (-0.00, 0.03) | | | 0.065 | 0.01 (-0.01, 0.02) | | | | 0.427 | 0.01 (0.00, 0.01) | | | **0.025** | 0.01 (0.00, 0.02) | | | | **0.037** | |
| **L5/S1** | 40 | 0.10 (-0.02, 0.21) | 0.100 | 0.06 (-0.05, 0.16) | | 0.278 | 0.00 (-0.01, 0.02) | | | 0.953 | 0.00 (-0.01, 0.02) | | | | 0.660 | 0.00 (-0.00, 0.01) | | | 0.297 | 0.01 (0.00, 0.02) | | | | **0.015** | |
| **Average spine (T11/T12-L5/S1)** | 40 | 0.09 (-0.01, 0.19) | 0.079 | 0.05 (-0.04, 0.14) | | 0.263 | 0.01 (0.00, 0.03) | | | **0.049** | 0.00 (-0.01, 0.02) | | | | 0.571 | 0.00 (-0.00, 0.01) | | | 0.102 | 0.01 (0.00, 0.02) | | | | 0.008 | |
| **All IVDs** | 280 | 0.09 (0.03, 0.15) | **0.004** | 0.05 (0.00, 0.11) | | **0.041** | 0.01 (0.01, 0.02) | | | **<0.001** | 0.00 (-0.00, 0.01) | | | | 0.298 | | 0.00 (0.00, 0.01) | | **0.011** | 0.01 (0.01, 0.02) | | | **<0.001** | |  |
| **Nucleus-to-annulus signal intensity ratio** | | | | | | | | |  | | |  | |  | | | | |  | |  | | | | |
| **T11/T12** | 40 | -0.02 (-0.05, -0.00) | **0.045** | -0.03 (-0.05, -0.01) | | **0.001** | -0.00 (-0.00, 0.00) | | | 0.521 | -0.00 (-0.00, 0.00) | | | | 0.850 | 0.00 (-0.00, 0.00) | | | 0.974 | -0.00 (-0.00, 0.00) | | | | 0.444 | |
| **T12/L1** | 40 | -0.01 (-0.03, 0.01) | 0.318 | -0.01 (-0.03, 0.01) | | 0.261 | 0.00 (-0.00, 0.00) | | | 0.627 | -0.00 (-0.00, 0.00) | | | | 0.407 | 0.00 (-0.00, 0.00) | | | 0.067 | -0.00 (-0.00, 0.00) | | | | 0.666 | |
| **L1/L2** | 40 | 0.01 (-0.01, 0.03) | 0.435 | -0.01 (-0.03, 0.02) | | 0.602 | 0.00 (0.00, 0.01) | | | **0.040** | 0.00 (-0.00, 0.00) | | | | 0.832 | -0.00 (-0.00, 0.00) | | | 0.770 | -0.00 (-0.00, 0.00) | | | | 0.972 | |
| **L2/L3** | 40 | -0.00 (-0.02, 0.02) | 0.817 | 0.00 (-0.02, 0.02) | | 0.956 | 0.00 (-0.00, 0.00) | | | 0.798 | 0.00 (-0.00, 0.00) | | | | 0.839 | 0.00 (-0.00, 0.00) | | | 0.784 | -0.00 (-0.00, 0.00) | | | | 0.119 | |
| **L3/L4** | 40 | -0.03 (-0.06, -0.01) | **0.007** | -0.02 (-0.04, 0.00) | | 0.072 | -0.00 (-0.01, 0.00) | | | 0.054 | 0.00 (-0.00, 0.01) | | | | 0.282 | 0.00 (-0.00, 0.00) | | | 0.858 | -0.00 (-0.00, 0.00) | | | | 0.609 | |
| **L4/L5** | 40 | -0.04 (-0.08, -0.01) | **0.024** | -0.01 (-0.04, 0.03) | | 0.633 | 0.00 (-0.01, 0.01) | | | 0.904 | 0.00 (-0.01, 0.01) | | | | 0.433 | -0.00 (-0.00, 0.00) | | | 0.714 | -0.00 (-0.00, 0.00) | | | | 0.832 | |
| **L5/S1** | 40 | -0.03 (-0.07, 0.01) | 0.092 | -0.01 (-0.05, 0.02) | | 0.378 | -0.00 (-0.01, 0.00) | | | 0.175 | -0.00 (-0.01, 0.00) | | | | 0.856 | -0.00 (-0.00, 0.00) | | | 0.808 | -0.00 (-0.00, 0.00) | | | | 0.484 | |
| **Average spine (T11/T12-L5/S1)** | 40 | -0.02 (-0.03, -0.00) | **0.010** | -0.01 (-0.02, 0.00) | | 0.067 | -0.00 (-0.00, 0.00) | | | 0.684 | 0.00 (-0.00, 0.00) | | | | 0.571 | 0.00 (-0.00, 0.00) | | | 0.699 | -0.00 (-0.00, 0.00) | | | | 0.439 | |
| **All IVDs** | 280 | -0.02 (-0.03, -0.01) | **0.001** | -0.01 (-0.02, -0.00) | | **0.012** | -0.00 (-0.00, 0.00) | | | 0.575 | 0.00 (-0.00, 0.00) | | | | 0.628 | 0.00 (-0.00, 0.00) | | | 0.833 | -0.00 (-0.00, 0.00) | | | | 0.183 | |
| **Pfirrmann grade** | | | | |  | | |  | |  | |  | |  | | | |  | |  | |  | | | |
| **T11/T12** | 40 | 0.02 (0.00, 0.04) | **0.039** | 0.01 (-0.00, 0.03) | | 0.103 | 0.00 (-0.00, 0.00) | | | 0.184 | 0.00 (-0.00, 0.00) | | | | 0.564 | -0.00 (-0.00, 0.00) | | | 0.522 | 0.00 (-0.00, 0.00) | | | | 0.489 | |
| **T12/L1** | 40 | -0.00 (-0.03, 0.03) | 0.988 | 0.01 (-0.01, 0.03) | | 0.332 | 0.00 (-0.00, 0.01) | | | 0.060 | 0.00 (-0.00, 0.01) | | | | 0.238 | -0.00 (-0.00, 0.00) | | | **0.030** | 0.00 (-0.00, 0.00) | | | | 0.574 | |
| **L1/L2** | 40 | -0.01 (-0.03, 0.01) | 0.370 | -0.01 (-0.03, 0.01) | | 0.557 | -0.00 (-0.00, 0.00) | | | 0.915 | 0.00 (-0.00, 0.00) | | | | 0.607 | -0.00 (-0.00, 0.00) | | | 0.612 | 0.00 (-0.00, 0.00) | | | | 0.557 | |
| **L2/L3** | 40 | 0.00 (-0.02, 0.02) | 0.921 | -0.01 (-0.02, 0.01) | | 0.602 | 0.00 (-0.00, 0.00) | | | 0.569 | 0.00 (-0.00, 0.00) | | | | 0.294 | 0.00 (-0.00, 0.00) | | | 0.808 | 0.00 (-0.00, 0.00) | | | | 0.323 | |
| **L3/L4** | 40 | 0.01 (-0.02, 0.04) | 0.516 | 0.00 (-0.03, 0.03) | | 0.980 | 0.00 (-0.00, 0.01) | | | 0.055 | 0.00 (-0.00, 0.01) | | | | 0.452 | 0.00 (-0.00, 0.00) | | | 0.957 | 0.00 (-0.00, 0.01) | | | | 0.907 | |
| **L4/L5** | 40 | 0.01 (-0.02, 0.03) | 0.717 | 0.01 (-0.01, 0.04) | | 0.336 | -0.00 (-0.01, 0.00) | | | 0.752 | -0.00 (-0.01, 0.00) | | | | 0.666 | 0.00 (-0.00, 0.00) | | | 0.935 | -0.00 (-0.00, 0.00) | | | | 0.535 | |
| **L5/S1** | 40 | 0.02 (-0.02, 0.06) | 0.218 | 0.01 (-0.02, 0.04) | | 0.568 | 0.01 (0.00, 0.01) | | | **0.019** | 0.00 (-0.01, 0.01) | | | | 0.136 | 0.00 (-0.00, 0.00) | | | 0.929 | 0.00 (-0.00, 0.01) | | | | 0.121 | |
| **Average spine (T11/T12-L5/S1)** | 40 | 0.01 (-0.00, 0.02) | 0.141 | 0.00 (-0.01, 0.01) | | 0.518 | 0.00 (-0.00, 0.00) | | | 0.129 | 0.00 (-0.00, 0.00) | | | | 0.261 | -0.00 (-0.00, 0.00) | | | 0.910 | 0.00 (0.00, 0.00) | | | | **0.026** | |
| **All IVDs** | 280 | 0.01 (-0.00, 0.02) | 0.235 | 0.00 (-0.01, 0.02) | | 0.327 | 0.00 (0.00, 0.01) | | | **0.006** | 0.00 (-0.00, 0.01) | | | | 0.071 | -0.00 (-0.00, 0.00) | | | 0.421 | 0.00 (-0.00, 0.00) | | | | 0.172 | |

Data are linear regression of independent variable total BPAQ score and dependent variable magnetic resonance IVD outcomes across all participants, males and females. Beta coefficient (β**)** indicate the relationship that moves in the same direction and variance as 95% confidence interval (95%CI). Pfirrmann grade is reverse scale where negative values indicate less degeneration. BPAQ: Bone-specific Physical Activity Questionnaire; Sex: biological sex; BMI: body mass index, IVD: Intervertebral disc; T2: time constant of the rate of proton relaxation in response to magnetization wither higher values indicating greater hydration.

**Table B.** Univariate linear regression of potential covariates with magnetic resonance IVD outcomes across male participants.

| **Potential covariate outcomes** | | | | | | | | | | | | | | | | | | | | | | | | |
| --- | --- | --- | --- | --- | --- | --- | --- | --- | --- | --- | --- | --- | --- | --- | --- | --- | --- | --- | --- | --- | --- | --- | --- | --- |
|  | | **Age** | | **BMI** | | | | **Current occupational sitting** | | | **Current occupational labour** | | | | | **Past occupational sitting** | | | **Past occupational labour** | | | | | |
|  | **n** | **β (CI)** | **P value** | **β (CI)** | | **P value** | **β (CI)** | | | **P value** | **β (CI)** | | | **P value** | **β (CI)** | | | **P value** | **β (CI)** | | | | **P value** | |
| **IVD T2 (ms)** | | | | | | | | | | | | | | | | | | | | | | | | |
| **T11/T12** | 20 | -0.76 (-1.85, 0.33) | 0.159 | -1.08 (-2.54, 0.38) | | 0.136 | -0.05 (-0.19, 0.08) | | | 0.432 | -0.01 (-0.14, 0.12) | | | 0.852 | 0.00 (-0.06, 0.06) | | | 0.921 | 0.02 (-0.07, 0.11) | | | | 0.618 | |
| **T12/L1** | 20 | -0.66 (-1.68, 0.36) | 0.188 | -1.66 (-2.85, -0.47) | | **0.009** | -0.04 (-0.16, 0.08) | | | 0.515 | -0.02 (-0.13, 0.09) | | | 0.688 | 0.02 (-0.04, 0.07) | | | 0.530 | 0.00 (-0.08, 0.08) | | | | 0.954 | |
| **L1/L2** | 20 | -0.73 (-1.69, 0.23) | 0.129 | -1.55 (-2.70, -0.40) | | **0.011** | -0.04 (-0.15, 0.06) | | | 0.398 | -0.01 (-0.11, 0.09) | | | 0.864 | 0.00 (-0.05, 0.05) | | | 0.957 | -0.01 (-0.08, 0.07) | | | | 0.825 | |
| **L2/L3** | 20 | -1.01 (-1.99, -0.02) | **0.046** | -1.16 (-2.54, 0.22) | | 0.095 | -0.07 (-0.19, 0.05) | | | 0.226 | 0.02 (-0.10, 0.13) | | | 0.761 | -0.00 (-0.06, 0.05) | | | 0.952 | 0.01 (-0.08, 0.09) | | | | 0.847 | |
| **L3/L4** | 20 | -1.07 (-2.11, -0.04) | **0.043** | -0.80 (-2.32, 0.72) | | 0.283 | -0.10 (-0.22, 0.03) | | | 0.134 | -0.02 (-0.15, 0.11) | | | 0.727 | 0.00 (-0.06, 0.06) | | | 0.950 | 0.03 (-0.07, 0.12) | | | | 0.528 | |
| **L4/L5** | 20 | -0.78 (-1.73, 0.17) | 0.103 | -1.30 (-2.53, -0.08) | | **0.039** | 0.03 (-0.06, 0.12) | | | 0.537 | 0.02 (-0.07, 0.10) | | | 0.707 | 0.01 (-0.03, 0.05) | | | 0.550 | 0.01 (-0.05, 0.07) | | | | 0.697 | |
| **L5/S1** | 20 | -1.23 (-2.41, -0.05) | **0.042** | -1.96 (-3.46, -0.45) | | **0.014** | -0.10 (-0.25, 0.04) | | | 0.156 | -0.03 (-0.18, 0.12) | | | 0.660 | 0.01 (-0.06, 0.08) | | | 0.815 | -0.02 (-0.13, 0.08) | | | | 0.639 | |
| **Average spine (T11/T12-L5/S1)** | 20 | -0.94 (-1.80, -0.08) | **0.033** | -1.18 (-2.37, 0.00) | | 0.051 | -0.03 (-0.14, 0.08) | | | 0.562 | 0.01 (-0.10, 0.11) | | | 0.875 | 0.00 (-0.05, 0.05) | | | 0.944 | 0.00 (-0.08, 0.08) | | | | 0.985 | |
| **All IVDs** | 140 | -0.89 (-1.25, -0.53) | **<0.001** | -1.36 (-1.84, -0.88) | | **<0.001** | -0.05 (-0.10, -0.01) | | | **0.013** | -0.01 (-0.05, 0.03) | | | 0.664 | 0.01 (-0.01, 0.02) | | | 0.582 | 0.01 (-0.02, 0.03) | | | | 0.699 | |
| **IVD height-to-vertebral body ratio** | | | | |  | | |  | |  | |  |  | | | |  | |  | |  | | | |
| **T11/T12** | 20 | -0.00 (-0.00, 0.00) | 0.552 | 0.00 (-0.00, 0.01) | | 0.098 | 0.00 (-0.00, 0.00) | | | 0.307 | -0.00 (-0.00, 0.00) | | | 0.689 | 0.00 (-0.00, 0.00) | | | 0.263 | 0.00 (-0.00, 0.00) | | | | 0.755 | |
| **T12/L1** | 20 | -0.00 (-0.00, 0.00) | 0.253 | -0.00 (-0.00, 0.00) | | 0.825 | 0.00 (-0.00, 0.00) | | | 0.518 | -0.00 (-0.00, 0.00) | | | 0.617 | 0.00 (-0.00, 0.00) | | | 0.238 | 0.00 (-0.00, 0.00) | | | | 0.786 | |
| **L1/L2** | 20 | -0.00 (-0.00, 0.00) | 0.404 | -0.00 (-0.00, 0.00) | | 0.850 | 0.00 (-0.00, 0.00) | | | 0.085 | -0.00 (-0.00, 0.00) | | | 0.894 | 0.00 (-0.00, 0.00) | | | 0.945 | -0.00 (-0.00, 0.00) | | | | 0.455 | |
| **L2/L3** | 20 | -0.00 (-0.00, 0.00) | 0.877 | -0.00 (-0.01, 0.00) | | 0.758 | 0.00 (-0.00, 0.00) | | | 0.359 | 0.00 (-0.00, 0.00) | | | 0.672 | 0.00 (-0.00, 0.00) | | | 0.538 | -0.00 (-0.00, 0.00) | | | | 0.872 | |
| **L3/L4** | 20 | -0.00 (-0.01, 0.00) | 0.737 | 0.00 (-0.01, 0.01) | | 0.636 | 0.00 (-0.00, 0.00) | | | 0.971 | 0.00 (-0.00, 0.00) | | | 0.619 | 0.00 (-0.00, 0.00) | | | 0.587 | 0.00 (-0.00, 0.00) | | | | 0.201 | |
| **L4/L5** | 20 | 0.00 (-0.00, 0.01) | 0.524 | -0.00 (-0.01, 0.00) | | 0.423 | -0.00 (-0.00, 0.00) | | | 0.833 | 0.00 (-0.00, 0.00) | | | 0.670 | 0.00 (0.00, 0.00) | | | 0.016 | -0.00 (-0.00, 0.00) | | | | 0.895 | |
| **L5/S1** | 20 | 0.00 (-0.00, 0.00) | 0.980 | -0.00 (-0.01, 0.00) | | 0.651 | -0.00 (-0.00, 0.00) | | | 0.150 | -0.00 (-0.00, 0.00) | | | 0.609 | 0.00 (-0.00, 0.00) | | | 0.304 | -0.00 (-0.00, 0.00) | | | | 0.961 | |
| **Average spine (T11/T12-L5/S1)** | 20 | -0.00 (-0.00, 0.00) | 0.540 | -0.00 (-0.00, 0.00) | | 0.799 | 0.00 (-0.00, 0.00) | | | 0.474 | 0.00 (-0.00, 0.00) | | | 0.908 | 0.00 (-0.00, 0.00) | | | 0.180 | 0.00 (-0.00, 0.00) | | | | 0.817 | |
| **All IVDs** | 140 | -0.00 (-0.00, 0.00) | 0.640 | -0.00 (-0.00, 0.00) | | 0.917 | 0.00 (-0.00, 0.00) | | | 0.607 | 0.00 (-0.00, 0.00) | | | 0.924 | 0.00 (0.00, 0.00) | | | **0.023** | 0.00 (-0.00, 0.00) | | | | 0.756 | |
| **IVD volume (cm^3)^** | | | | | |  |  | | |  |  | | |  |  | | |  |  | | | |  | |
| **T11/T12** | 20 | -0.03 (-0.16, 0.10) | 0.648 | 0.14 (-0.01, 0.30) | | 0.070 | 0.01 (-0.01, 0.02) | | | 0.297 | 0.00 (-0.01, 0.02) | | | 0.686 | 0.00 (-0.01, 0.01) | | | 0.533 | 0.00 (-0.01, 0.01) | | | | 0.505 | |
| **T12/L1** | 20 | -0.09 (-0.23, 0.06) | 0.225 | 0.09 (-0.11, 0.28) | | 0.365 | 0.00 (-0.02, 0.02) | | | 0.792 | -0.00 (-0.02, 0.02) | | | 0.897 | -0.00 (-0.01, 0.01) | | | 0.719 | 0.01 (-0.01, 0.02) | | | | 0.337 | |
| **L1/L2** | 20 | -0.13 (-0.34, 0.07) | 0.187 | 0.05 (-0.24, 0.33) | | 0.737 | 0.01 (-0.01, 0.03) | | | 0.449 | 0.00 (-0.02, 0.02) | | | 0.965 | -0.00 (-0.01, 0.01) | | | 0.417 | 0.00 (-0.01, 0.02) | | | | 0.675 | |
| **L2/L3** | 20 | -0.08 (-0.28, 0.13) | 0.442 | -0.00 (-0.28, 0.28) | | 0.999 | 0.01 (-0.02, 0.03) | | | 0.614 | 0.00 (-0.02, 0.02) | | | 0.964 | -0.00 (-0.01, 0.01) | | | 0.631 | 0.00 (-0.01, 0.02) | | | | 0.745 | |
| **L3/L4** | 20 | -0.08 (-0.33, 0.16) | 0.487 | 0.14 (-0.19, 0.47) | | 0.385 | 0.00 (-0.03, 0.03) | | | 0.965 | 0.01 (-0.02, 0.03) | | | 0.693 | -0.00 (-0.02, 0.01) | | | 0.521 | 0.02 (-0.00, 0.03) | | | | 0.101 | |
| **L4/L5** | 20 | -0.00 (-0.17, 0.17) | 0.998 | 0.06 (-0.17, 0.30) | | 0.571 | -0.00 (-0.02, 0.02) | | | 0.992 | 0.00 (-0.01, 0.02) | | | 0.696 | 0.00 (-0.00, 0.01) | | | 0.377 | 0.01 (-0.01, 0.02) | | | | 0.380 | |
| **L5/S1** | 20 | 0.00 (-0.14, 0.15) | 0.962 | 0.10 (-0.09, 0.28) | | 0.292 | -0.01 (-0.03, 0.00) | | | 0.075 | -0.00 (-0.02, 0.01) | | | 0.767 | 0.00 (-0.01, 0.01) | | | 0.874 | 0.01 (-0.00, 0.02) | | | | 0.107 | |
| **Average spine (T11/T12-L5/S1)** | 20 | -0.07 (-0.20, 0.06) | 0.282 | 0.05 (-0.13, 0.23) | | 0.553 | -0.00 (-0.02, 0.01) | | | 0.922 | 0.00 (-0.01, 0.02) | | | 0.897 | -0.00 (-0.01, 0.01) | | | 0.881 | 0.01 (-0.00, 0.02) | | | | 0.208 | |
| **All IVDs** | 140 | -0.06 (-0.15, 0.03) | 0.209 | 0.08 (-0.04, 0.20) | | 0.181 | 0.00 (-0.01, 0.01) | | | 0.745 | 0.00 (-0.01, 0.01) | | | 0.774 | | -0.00 (-0.01, 0.01) | | 0.721 | 0.01 (-0.00, 0.01) | | | 0.068 | |  |
| **Nucleus-to-annulus signal intensity ratio** | | | | | | | | |  | | |  |  | | | | |  | |  | | | | |
| **T11/T12** | 20 | -0.01 (-0.05, 0.03) | 0.673 | -0.03 (-0.08, 0.03) | | 0.297 | -0.00 (-0.01, 0.00) | | | 0.903 | -0.00 (-0.00, 0.00) | | | 0.855 | 0.00 (-0.00, 0.00) | | | 0.290 | -0.00 (-0.00, 0.00) | | | | 0.531 | |
| **T12/L1** | 20 | -0.02 (-0.06, 0.02) | 0.265 | -0.02 (-0.07, 0.03) | | 0.414 | -0.00 (-0.00, 0.00) | | | 0.988 | -0.00 (-0.01, 0.00) | | | 0.595 | 0.00 (-0.00, 0.00) | | | 0.061 | -0.00 (-0.00, 0.00) | | | | 0.661 | |
| **L1/L2** | 20 | -0.02 (-0.05, 0.01) | 0.286 | -0.01 (-0.05, 0.04) | | 0.799 | 0.00 (-0.00, 0.01) | | | 0.078 | 0.00 (-0.00, 0.00) | | | 0.938 | -0.00 (-0.00, 0.00) | | | 0.533 | -0.00 (-0.00, 0.00) | | | | 0.624 | |
| **L2/L3** | 20 | -0.02 (-0.05, 0.01) | 0.150 | -0.02 (-0.06, 0.02) | | 0.337 | 0.00 (-0.00, 0.00) | | | 0.506 | 0.00 (-0.00, 0.00) | | | 0.735 | 0.00 (-0.00, 0.00) | | | 0.670 | -0.00 (-0.00, 0.00) | | | | 0.428 | |
| **L3/L4** | 20 | -0.03 (-0.07, 0.01) | 0.118 | -0.03 (-0.09, 0.02) | | 0.198 | -0.00 (-0.01, 0.00) | | | 0.395 | 0.00 (-0.00, 0.01) | | | 0.267 | 0.00 (-0.00, 0.00) | | | 0.239 | 0.00 (-0.00, 0.00) | | | | 0.449 | |
| **L4/L5** | 20 | -0.02 (-0.07, 0.03) | 0.501 | -0.01 (-0.08, 0.06) | | 0.699 | 0.00 (-0.00, 0.01) | | | 0.188 | 0.00 (-0.00, 0.01) | | | 0.242 | 0.00 (-0.00, 0.00) | | | 0.564 | 0.00 (-0.00, 0.00) | | | | 0.983 | |
| **L5/S1** | 20 | -0.04 (-0.11, 0.02) | 0.147 | -0.03 (-0.12, 0.05) | | 0.431 | -0.00 (-0.01, 0.01) | | | 0.284 | -0.00 (-0.01, 0.01) | | | 0.711 | 0.00 (-0.00, 0.01) | | | 0.650 | -0.00 (-0.01, 0.01) | | | | 0.488 | |
| **Average spine (T11/T12-L5/S1)** | 20 | -0.02 (-0.04, 0.00) | 0.096 | -0.02 (-0.05, 0.01) | | 0.182 | -0.00 (-0.00, 0.00) | | | 0.970 | 0.00 (-0.00, 0.00) | | | 0.664 | 0.00 (-0.00, 0.00) | | | 0.204 | -0.00 (-0.00, 0.00) | | | | 0.717 | |
| **All IVDs** | 140 | -0.02 (-0.04, -0.01) | **0.007** | -0.02 (-0.04, 0.00) | | 0.057 | 0.00 (-0.00, 0.00) | | | 0.790 | 0.00 (-0.00, 0.00) | | | 0.592 | 0.00 (-0.00, 0.00) | | | 0.074 | -0.00 (-0.00, 0.00) | | | | 0.423 | |
| **Pfirrmann grade** | | | | |  | | |  | |  | |  |  | | | |  | |  | |  | | | |
| **T11/T12** | 20 | 0.01 (-0.02, 0.05) | 0.336 | 0.02 (-0.03, 0.06) | | 0.477 | 0.00 (-0.00, 0.01) | | | 0.376 | 0.00 (-0.00, 0.00) | | | 0.791 | -0.00 (-0.00, 0.00) | | | 0.354 | 0.00 (-0.00, 0.00) | | | | 0.657 | |
| **T12/L1** | 20 | 0.00 (-0.05, 0.05) | 0.944 | 0.05 (-0.01, 0.11) | | 0.113 | 0.00 (-0.00, 0.01) | | | 0.076 | 0.00 (-0.01, 0.01) | | | 0.646 | -0.00 (-0.00, 0.00) | | | 0.077 | 0.00 (-0.00, 0.01) | | | | 0.728 | |
| **L1/L2** | 20 | 0.01 (-0.02, 0.04) | 0.653 | 0.03 (-0.01, 0.06) | | 0.185 | -0.00 (-0.00, 0.00) | | | 0.576 | 0.00 (-0.00, 0.00) | | | 0.488 | 0.00 (-0.00, 0.00) | | | 0.637 | 0.00 (-0.00, 0.00) | | | | 0.567 | |
| **L2/L3** | 20 | 0.01 (-0.02, 0.04) | 0.653 | 0.03 (-0.01, 0.06) | | 0.185 | -0.00 (-0.00, 0.00) | | | 0.576 | 0.00 (-0.00, 0.00) | | | 0.488 | 0.00 (-0.00, 0.00) | | | 0.637 | 0.00 (-0.00, 0.00) | | | | 0.567 | |
| **L3/L4** | 20 | 0.01 (-0.04, 0.06) | 0.800 | 0.01 (-0.05, 0.08) | | 0.660 | 0.00 (-0.01, 0.01) | | | 0.457 | 0.00 (-0.01, 0.01) | | | 0.786 | -0.00 (-0.00, 0.01) | | | 0.638 | -0.00 (-0.01, 0.01) | | | | 0.274 | |
| **L4/L5** | 20 | -0.02 (-0.05, 0.02) | 0.304 | 0.02 (-0.02, 0.07) | | 0.317 | -0.00 (-0.01, 0.00) | | | 0.540 | -0.00 (-0.00, 0.00) | | | 0.389 | -0.00 (-0.00, 0.00) | | | 0.468 | -0.00 (-0.01, 0.00) | | | | 0.128 | |
| **L5/S1** | 20 | 0.01 (-0.05, 0.06) | 0.807 | 0.05 (-0.02, 0.12) | | 0.170 | 0.00 (-0.01, 0.01) | | | 0.163 | 0.00 (-0.01, 0.01) | | | 0.150 | -0.00 (-0.01, 0.01) | | | 0.242 | 0.00 (-0.01, 0.01) | | | | 0.285 | |
| **Average spine (T11/T12-L5/S1)** | 20 | 0.01 (-0.01, 0.02) | 0.501 | 0.02 (0.00, 0.04) | | **0.019** | 0.00 (-0.00, 0.00) | | | 0.758 | 0.00 (-0.00, 0.00) | | | 0.614 | -0.00 (-0.00, 0.00) | | | 0.121 | 0.00 (-0.00, 0.00) | | | | 0.138 | |
| **All IVDs** | 140 | 0.00 (-0.01, 0.02) | 0.684 | 0.03 (0.01, 0.05) | | **0.011** | 0.00 (-0.00, 0.01) | | | 0.128 | 0.00 (-0.00, 0.01) | | | 0.249 | -0.00 (-0.00, 0.00) | | | 0.087 | 0.00 (-0.00, 0.01) | | | | 0.831 | |

Data are linear regression of independent variable total BPAQ score and dependent variable magnetic resonance IVD outcomes across all participants, males and females. Beta coefficient (β**)** indicate the relationship that moves in the same direction and variance as 95% confidence interval (95%CI). Pfirrmann grade is reverse scale where negative values indicate less degeneration. BPAQ: Bone-specific Physical Activity Questionnaire; Sex: biological sex; BMI: body mass index, IVD: Intervertebral disc; T2: time constant of the rate of proton relaxation in response to magnetization wither higher values indicating greater hydration. **Table C.** Univariate linear regression of potential covariates with magnetic resonance IVD outcomes across female participants.

| **Potential covariate outcomes** | | | | | | | | | | | | | | | | | | | | | | | | |
| --- | --- | --- | --- | --- | --- | --- | --- | --- | --- | --- | --- | --- | --- | --- | --- | --- | --- | --- | --- | --- | --- | --- | --- | --- |
|  | | **Age** | | **BMI** | | | **Current occupational sitting** | | | | | **Current occupational labour** | | | | **Past occupational sitting** | | | **Past occupational labour** | | | | | |
|  | **n** | **β (CI)** | **P value** | **β (CI)** | | **P value** | **β (CI)** | | **P value** | **β (CI)** | | | | **P value** | **β (CI)** | | | **P value** | **β (CI)** | | | | **P value** | |
| **IVD T2 (ms)** | | | | | | | | | | | | | | | | | | | | | | | | |
| **T11/T12** | 20 | -0.96 (-1.68, -0.23) | **0.013** | -0.57 (-1.02, -0.13) | | **0.014** | -0.12 (-0.26, 0.03) | | 0.101 | -0.00 (-0.20, 0.19) | | | | 0.982 | -0.04 (-0.09, 0.01) | | | 0.145 | -0.30 (-0.64, 0.04) | | | | 0.077 | |
| **T12/L1** | 20 | -0.38 (-0.99, 0.24) | 0.213 | -0.39 (-0.72, -0.05) | | **0.027** | 0.00 (-0.11, 0.12) | | 0.969 | -0.03 (-0.17, 0.11) | | | | 0.647 | -0.01 (-0.05, 0.03) | | | 0.597 | -0.16 (-0.42, 0.10) | | | | 0.219 | |
| **L1/L2** | 20 | 0.03 (-0.72, 0.79) | 0.926 | -0.31 (-0.74, 0.12) | | 0.150 | 0.01 (-0.12, 0.15) | | 0.840 | 0.03 (-0.14, 0.19) | | | | 0.756 | -0.02 (-0.07, 0.03) | | | 0.340 | -0.24 (-0.54, 0.06) | | | | 0.115 | |
| **L2/L3** | 20 | -0.18 (-0.88, 0.52) | 0.587 | -0.35 (-0.74, 0.04) | | 0.073 | 0.04 (-0.08, 0.17) | | 0.460 | 0.06 (-0.10, 0.21) | | | | 0.438 | -0.00 (-0.05, 0.04) | | | 0.955 | -0.18 (-0.47, 0.11) | | | | 0.213 | |
| **L3/L4** | 20 | -0.39 (-0.98, 0.19) | 0.175 | -0.38 (-0.70, -0.06) | | **0.024** | -0.05 (-0.15, 0.06) | | 0.375 | 0.05 (-0.08, 0.19) | | | | 0.407 | -0.03 (-0.07, 0.01) | | | 0.099 | -0.15 (-0.40, 0.11) | | | | 0.239 | |
| **L4/L5** | 20 | -1.24 (-2.05, -0.43) | **0.005** | -0.41 (-0.99, 0.17) | | 0.157 | -0.08 (-0.26, 0.09) | | 0.346 | -0.01 (-0.24, 0.22) | | | | 0.915 | -0.07 (-0.13, -0.01) | | | **0.019** | -0.05 (-0.48, 0.39) | | | | 0.821 | |
| **L5/S1** | 20 | -0.38 (-1.43, 0.67) | 0.454 | -0.13 (-0.78, 0.51) | | 0.665 | 0.05 (-0.14, 0.24) | | 0.596 | -0.09 (-0.32, 0.15) | | | | 0.453 | -0.02 (-0.09, 0.04) | | | 0.467 | 0.20 (-0.25, 0.64) | | | | 0.368 | |
| **Average spine (T11/T12-L5/S1)** | 20 | -0.54 (-1.05, -0.02) | **0.041** | -0.36 (-0.66, -0.05) | | **0.023** | -0.01 (-0.12, 0.09) | | 0.787 | -0.02 (-0.14, 0.11) | | | | 0.808 | -0.03 (-0.06, 0.00) | | | 0.086 | -0.14 (-0.38, 0.10) | | | | 0.240 | |
| **All IVDs** | 140 | -0.50 (-0.78, -0.22) | **0.001** | -0.36 (-0.53, -0.20) | | **<0.001** | -0.02 (-0.07, 0.03) | | 0.459 | 0.00 (-0.06, 0.07) | | | | 0.974 | -0.03 (-0.05, -0.01) | | | **0.003** | -0.13 (-0.25, -0.00) | | | | **0.048** | |
| **IVD height-to-vertebral body ratio** | | | | |  | |  | |  | |  | |  | | | |  | |  | |  | | | |
| **T11/T12** | 20 | -0.00 (-0.00, 0.00) | 0.969 | -0.00 (-0.00, 0.00) | | 0.729 | 0.00 (-0.00, 0.00) | | 0.387 | 0.00 (-0.00, 0.00) | | | | 0.266 | 0.00 (-0.00, 0.00) | | | 0.348 | -0.00 (-0.00, 0.00) | | | | 0.709 | |
| **T12/L1** | 20 | 0.00 (-0.00, 0.00) | 0.266 | 0.00 (-0.00, 0.00) | | 0.155 | 0.00 (0.00, 0.00) | | **0.033** | -0.00 (-0.00, 0.00) | | | | 0.867 | 0.00 (-0.00, 0.00) | | | 0.056 | 0.00 (-0.00, 0.00) | | | | 0.279 | |
| **L1/L2** | 20 | 0.00 (0.00, 0.01) | **0.021** | 0.00 (-0.00, 0.00) | | 0.116 | 0.00 (-0.00, 0.00) | | 0.132 | 0.00 (-0.00, 0.00) | | | | 0.222 | 0.00 (-0.00, 0.00) | | | 0.074 | 0.00 (-0.00, 0.00) | | | | 0.735 | |
| **L2/L3** | 20 | 0.00 (0.00, 0.01) | **0.033** | 0.00 (-0.00, 0.00) | | 0.262 | 0.00 (-0.00, 0.00) | | 0.402 | 0.00 (-0.00, 0.00) | | | | 0.284 | 0.00 (-0.00, 0.00) | | | 0.333 | -0.00 (-0.00, 0.00) | | | | 0.668 | |
| **L3/L4** | 20 | 0.00 (0.00, 0.01) | **0.020** | 0.00 (-0.00, 0.00) | | 0.275 | 0.00 (-0.00, 0.00) | | 0.409 | 0.00 (-0.00, 0.00) | | | | 0.681 | 0.00 (-0.00, 0.00) | | | 0.176 | -0.00 (-0.00, 0.00) | | | | 0.579 | |
| **L4/L5** | 20 | 0.00 (-0.00, 0.00) | 0.343 | 0.00 (-0.00, 0.00) | | 0.678 | 0.00 (-0.00, 0.00) | | 0.393 | 0.00 (-0.00, 0.00) | | | | 0.518 | 0.00 (-0.00, 0.00) | | | 0.978 | 0.00 (-0.00, 0.00) | | | | 0.839 | |
| **L5/S1** | 20 | -0.00 (-0.01, 0.00) | 0.509 | 0.00 (-0.00, 0.00) | | 0.797 | -0.00 (-0.00, -0.00) | | **0.041** | 0.00 (-0.00, 0.00) | | | | 0.919 | -0.00 (-0.00, 0.00) | | | 0.182 | -0.00 (-0.00, 0.00) | | | | 0.540 | |
| **Average spine (T11/T12-L5/S1)** | 20 | 0.00 (-0.00, 0.00) | 0.146 | 0.00 (-0.00, 0.00) | | 0.340 | 0.00 (-0.00, 0.00) | | 0.331 | 0.00 (-0.00, 0.00) | | | | 0.867 | 0.00 (-0.00, 0.00) | | | 0.483 | -0.00 (-0.00, 0.00) | | | | 0.618 | |
| **All IVDs** | 140 | 0.00 (-0.00, 0.00) | 0.072 | 0.00 (-0.00, 0.00) | | 0.158 | 0.00 (-0.00, 0.00) | | 0.463 | 0.00 (-0.00, 0.00) | | | | 0.271 | 0.00 (-0.00, 0.00) | | | 0.309 | -0.00 (-0.00, 0.00) | | | | **<0.001** | |
| **IVD volume (cm^3)^** | | | | | |  |  | |  |  | | | |  |  | | |  |  | | | |  | |
| **T11/T12** | 20 | 0.06 (-0.04, 0.16) | 0.238 | 0.01 (-0.05, 0.08) | | 0.667 | 0.01 (-0.01, 0.03) | | 0.318 | -0.00 (-0.03, 0.02) | | | | 0.811 | 0.00 (-0.00, 0.01) | | | 0.281 | -0.01 (-0.06, 0.03) | | | | 0.582 | |
| **T12/L1** | 20 | 0.09 (-0.04, 0.21) | 0.171 | 0.05 (-0.03, 0.13) | | 0.212 | 0.01 (-0.02, 0.03) | | 0.562 | -0.01 (-0.04, 0.02) | | | | 0.603 | 0.01 (-0.00, 0.01) | | | 0.176 | -0.01 (-0.06, 0.05) | | | | 0.829 | |
| **L1/L2** | 20 | 0.14 (-0.00, 0.28) | 0.055 | 0.06 (-0.03, 0.15) | | 0.200 | 0.02 (-0.01, 0.05) | | 0.138 | 0.01 (-0.03, 0.04) | | | | 0.700 | 0.01 (0.00, 0.02) | | | **0.030** | -0.01 (-0.07, 0.06) | | | | 0.858 | |
| **L2/L3** | 20 | 0.15 (-0.04, 0.34) | 0.119 | 0.10 (-0.02, 0.21) | | 0.093 | 0.01 (-0.02, 0.05) | | 0.474 | -0.01 (-0.05, 0.04) | | | | 0.691 | 0.01 (-0.00, 0.02) | | | 0.129 | -0.02 (-0.11, 0.07) | | | | 0.672 | |
| **L3/L4** | 20 | 0.19 (-0.03, 0.42) | 0.081 | 0.11 (-0.02, 0.25) | | 0.103 | 0.03 (-0.01, 0.07) | | 0.158 | -0.01 (-0.07, 0.04) | | | | 0.615 | 0.01 (0.00, 0.03) | | | **0.045** | 0.01 (-0.09, 0.11) | | | | 0.839 | |
| **L4/L5** | 20 | 0.14 (-0.03, 0.32) | 0.102 | 0.07 (-0.04, 0.18) | | 0.216 | 0.02 (-0.01, 0.05) | | 0.167 | 0.00 (-0.04, 0.04) | | | | 0.882 | 0.01 (-0.00, 0.02) | | | 0.084 | -0.00 (-0.08, 0.08) | | | | 0.980 | |
| **L5/S1** | 20 | 0.07 (-0.12, 0.26) | 0.454 | 0.06 (-0.05, 0.18) | | 0.270 | -0.01 (-0.05, 0.02) | | 0.467 | 0.01 (-0.03, 0.05) | | | | 0.603 | 0.00 (-0.01, 0.01) | | | 0.721 | -0.03 (-0.11, 0.06) | | | | 0.504 | |
| **Average spine (T11/T12-L5/S1)** | 20 | 0.14 (0.00, 0.27) | **0.047** | 0.07 (-0.02, 0.15) | | 0.109 | 0.01 (-0.01, 0.04) | | 0.239 | -0.00 (-0.04, 0.03) | | | | 0.912 | 0.01 (0.00, 0.02) | | | 0.054 | -0.00 (-0.07, 0.06) | | | | 0.883 | |
| **All IVDs** | 140 | 0.12 (0.04, 0.20) | **0.005** | 0.07 (0.01, 0.12) | | **0.013** | 0.01 (-0.00, 0.03) | | 0.112 | -0.00 (-0.02, 0.02) | | | | 0.866 | | 0.01 (0.00, 0.01) | | **0.005** | -0.01 (-0.05, 0.03) | | | 0.648 | |  |
| **Nucleus-to-annulus signal intensity ratio** | | | | | | | |  | | |  | |  | | | | |  | |  | | | | |
| **T11/T12** | 20 | -0.05 (-0.08, -0.03) | **0.001** | -0.03 (-0.05, -0.02) | | **0.001** | -0.01 (-0.01, 0.00) | | 0.111 | -0.00 (-0.01, 0.01) | | | | 0.906 | -0.00 (-0.01, 0.00) | | | 0.053 | -0.02 (-0.03, -0.00) | | | | **0.020** | |
| **T12/L1** | 20 | -0.01 (-0.03, 0.02) | 0.681 | -0.01 (-0.02, 0.01) | | 0.369 | 0.00 (-0.00, 0.01) | | 0.253 | -0.00 (-0.01, 0.01) | | | | 0.440 | -0.00 (-0.00, 0.00) | | | 0.925 | -0.00 (-0.01, 0.01) | | | | 0.667 | |
| **L1/L2** | 20 | 0.03 (-0.02, 0.07) | 0.225 | -0.00 (-0.03, 0.02) | | 0.766 | 0.00 (-0.01, 0.01) | | 0.477 | -0.00 (-0.01, 0.01) | | | | 0.966 | -0.00 (-0.00, 0.00) | | | 0.815 | -0.01 (-0.03, 0.01) | | | | 0.166 | |
| **L2/L3** | 20 | 0.03 (0.00, 0.06) | **0.040** | 0.00 (-0.01, 0.02) | | 0.645 | 0.00 (-0.00, 0.01) | | 0.314 | 0.00 (-0.01, 0.01) | | | | 0.771 | 0.00 (-0.00, 0.00) | | | 0.558 | -0.00 (-0.02, 0.01) | | | | 0.646 | |
| **L3/L4** | 20 | -0.03 (-0.06, 0.01) | 0.135 | -0.02 (-0.04, 0.00) | | 0.086 | -0.00 (-0.01, 0.00) | | 0.612 | 0.00 (-0.00, 0.01) | | | | 0.339 | -0.00 (-0.00, 0.00) | | | 0.569 | -0.01 (-0.03, 0.00) | | | | 0.158 | |
| **L4/L5** | 20 | -0.08 (-0.14, -0.01) | **0.023** | -0.01 (-0.05, 0.04) | | 0.712 | -0.00 (-0.02, 0.01) | | 0.484 | 0.00 (-0.02, 0.02) | | | | 0.948 | -0.00 (-0.01, 0.00) | | | 0.373 | 0.01 (-0.02, 0.04) | | | | 0.461 | |
| **L5/S1** | 20 | -0.03 (-0.09, 0.03) | 0.280 | -0.01 (-0.05, 0.03) | | 0.563 | -0.01 (-0.02, 0.00) | | 0.208 | 0.00 (-0.01, 0.02) | | | | 0.672 | -0.00 (-0.01, 0.01) | | | 0.186 | -0.01 (-0.04, 0.01) | | | | 0.306 | |
| **Average spine (T11/T12-L5/S1)** | 20 | -0.02 (-0.03, -0.00) | **0.021** | -0.01 (-0.02, 0.00) | | 0.103 | -0.00 (-0.00, 0.00) | | 0.709 | 0.00 (-0.00, 0.00) | | | | 0.653 | -0.00 (-0.00, 0.00) | | | 0.070 | -0.00 (-0.01, 0.00) | | | | 0.286 | |
| **All IVDs** | 140 | -0.02 (-0.04, -0.00) | **0.035** | -0.01 (-0.02, 0.00) | | 0.056 | -0.00 (-0.01, 0.00) | | 0.416 | 0.00 (-0.00, 0.00) | | | | 0.732 | -0.00 (-0.00, 0.00) | | | 0.088 | -0.01 (-0.02, 0.00) | | | | 0.091 | |
| **Pfirrmann grade** | | | | |  | |  | |  | |  | |  | | | |  | |  | |  | | | |
| **T11/T12** | 20 | 0.03 (0.00, 0.05) | **0.039** | 0.01 (-0.00, 0.03) | | 0.096 | 0.00 (-0.00, 0.01) | | 0.358 | 0.00 (-0.00, 0.01) | | | | 0.481 | 0.00 (-0.00, 0.00) | | | 0.690 | 0.00 (-0.01, 0.02) | | | | 0.539 | |
| **T12/L1** | 20 | -0.00 (-0.04, 0.03) | 0.884 | 0.00 (-0.02, 0.02) | | 0.832 | 0.00 (-0.01, 0.01) | | 0.906 | 0.01 (0.00, 0.01) | | | | **0.044** | -0.00 (-0.00, 0.00) | | | 0.286 | 0.00 (-0.01, 0.02) | | | | 0.745 | |
| **L1/L2** | 20 | -0.03 (-0.07, 0.01) | 0.103 | -0.01 (-0.04, 0.01) | | 0.297 | 0.00 (-0.01, 0.01) | | 0.747 | -0.00 (-0.01, 0.01) | | | | 0.948 | -0.00 (-0.01, 0.00) | | | 0.169 | 0.01 (-0.01, 0.02) | | | | 0.562 | |
| **L2/L3** | 20 | -0.02 (-0.06, 0.02) | 0.408 | -0.01 (-0.04, 0.01) | | 0.360 | 0.00 (-0.00, 0.01) | | 0.465 | 0.00 (-0.01, 0.01) | | | | 0.443 | -0.00 (-0.00, 0.00) | | | 0.542 | -0.00 (-0.02, 0.02) | | | | 0.902 | |
| **L3/L4** | 20 | -0.01 (-0.05, 0.04) | 0.657 | -0.00 (-0.03, 0.03) | | 0.986 | 0.00 (-0.00, 0.01) | | 0.382 | 0.00 (-0.01, 0.01) | | | | 0.594 | -0.00 (-0.00, 0.00) | | | 0.938 | 0.01 (-0.01, 0.03) | | | | 0.156 | |
| **L4/L5** | 20 | 0.03 (-0.02, 0.08) | 0.243 | 0.01 (-0.02, 0.04) | | 0.561 | -0.00 (-0.01, 0.01) | | 0.569 | 0.00 (-0.01, 0.01) | | | | 0.884 | 0.00 (-0.00, 0.00) | | | 0.595 | 0.00 (-0.02, 0.02) | | | | 0.978 | |
| **L5/S1** | 20 | 0.04 (-0.03, 0.11) | 0.270 | 0.00 (-0.04, 0.05) | | 0.921 | 0.01 (0.00, 0.02) | | 0.050 | 0.00 (-0.01, 0.02) | | | | 0.793 | 0.00 (-0.00, 0.01) | | | 0.167 | 0.03 (-0.00, 0.05) | | | | 0.077 | |
| **Average spine (T11/T12-L5/S1)** | 20 | 0.01 (-0.01, 0.03) | 0.457 | -0.00 (-0.01, 0.01) | | 0.879 | 0.00 (-0.00, 0.01) | | 0.226 | 0.00 (-0.00, 0.01) | | | | 0.268 | 0.00 (-0.00, 0.00) | | | 0.259 | 0.01 (-0.00, 0.01) | | | | 0.153 | |
| **All IVDs** | 140 | 0.00 (-0.01, 0.02) | 0.627 | 0.00 (-0.01, 0.01) | | 0.964 | 0.00 (-0.00, 0.01) | | 0.112 | 0.00 (-0.00, 0.01) | | | | 0.226 | 0.00 (-0.00, 0.00) | | | 0.920 | 0.01 (-0.00, 0.01) | | | | 0.085 | |

Data are linear regression of independent variable total BPAQ score and dependent variable magnetic resonance IVD outcomes across all participants, males and females. Beta coefficient (β**)** indicate the relationship that moves in the same direction and variance as 95% confidence interval (95%CI). Pfirrmann grade is reverse scale where negative values indicate less degeneration. BPAQ: Bone-specific Physical Activity Questionnaire; Sex: biological sex; BMI: body mass index, IVD: Intervertebral disc; T2: time constant of the rate of proton relaxation in response to magnetization wither higher values indicating greater hydration.

**Table D.** Univariate linear regression of potential covariates with magnetic resonance IVD T2 across five IVD regions.

| **Potential covariate factors** | | | | | | | | | | | | | | | | | | | | | | | | | | | | | | |  |
| --- | --- | --- | --- | --- | --- | --- | --- | --- | --- | --- | --- | --- | --- | --- | --- | --- | --- | --- | --- | --- | --- | --- | --- | --- | --- | --- | --- | --- | --- | --- | --- |
|  | | **Age** | | **BMI** | | | | | **Current occupational sitting** | | | | | **Current occupational labour** | | | | | **Past occupational sitting** | | | | **Past occupational labour** | | | | | | | |  |
|  | **n** | **β (CI)** | **P value** | **β (CI)** | | | **P value** | **β (CI)** | | | **P value** | **β (CI)** | | | | | **P value** | **β (CI)** | | | | **P value** | **β (CI)** | | | | | | **P value** | |  |
| **Region 1 IVD T2 (ms)** | | | | | | | | | | | | | | | | | | | | | | | | | | | | | | |  |
| **T11/T12** | 40 | 0.07 (-0.41, 0.56) | 0.761 | 0.26 (-0.15, 0.68) | | | 0.204 | -0.00 (-0.06, 0.06) | | | 0.973 | 0.00 (-0.07, 0.07) | | | | | 0.899 | -0.00 (-0.04, 0.02) | | | | 0.533 | 0.05 (0.01, 0.10) | | | | | | **0.017** | |  |
| **T12/L1** | 40 | 0.14 (-0.33, 0.62) | 0.546 | 0.02 (-0.39, 0.44) | | | 0.910 | -0.01 (-0.08, 0.05) | | | 0.662 | 0.02 (-0.05, 0.08) | | | | | 0.625 | -0.01 (-0.04, 0.02) | | | | 0.531 | 0.03 (-0.02, 0.07) | | | | | | 0.215 | |  |
| **L1/L2** | 40 | -0.11 (-0.69, 0.46) | 0.694 | 0.03 (-0.47, 0.53) | | | 0.908 | -0.02 (-0.09, 0.05) | | | 0.573 | 0.00 (-0.08, 0.08) | | | | | 0.958 | -0.02 (-0.05, 0.01) | | | | 0.261 | 0.03 (-0.02, 0.09) | | | | | | 0.243 | |  |
| **L2/L3** | 40 | -0.15 (-0.69, 0.39) | 0.584 | -0.22 (-0.68, 0.24) | | | 0.341 | -0.03 (-0.10, 0.03) | | | 0.330 | 0.03 (-0.04, 0.10) | | | | | 0.376 | 0.01 (-0.02, 0.04) | | | | 0.356 | 0.02 (-0.03, 0.07) | | | | | | 0.489 | |  |
| **L3/L4** | 40 | -0.41 (-1.09, 0.28) | 0.238 | -0.38 (-0.97, 0.21) | | | 0.204 | -0.05 (-0.14, 0.04) | | | 0.250 | 0.00 (-0.10, 0.10) | | | | | 0.960 | -0.01 (-0.05, 0.03) | | | | 0.750 | 0.03 (-0.04, 0.09) | | | | | | 0.429 | |  |
| **L4/L5** | 40 | -0.44 (-0.89, 0.00) | 0.052 | -0.40 (-0.79, -0.02) | | | **0.041** | -0.01 (-0.07, 0.04) | | | 0.625 | -0.03 (-0.09, 0.03) | | | | | 0.357 | -0.01 (-0.03, 0.02) | | | | 0.518 | -0.03 (-0.07, 0.01) | | | | | | 0.180 | |  |
| **L5/S1** | 40 | -0.87 (-1.55, -0.19) | **0.013** | -0.44 (-1.06, 0.18) | | | 0.156 | -0.06 (-0.16, 0.03) | | | 0.161 | -0.06 (-0.16, 0.04) | | | | | 0.260 | -0.01 (-0.05, 0.03) | | | | 0.657 | -0.04 (-0.11, 0.03) | | | | | | 0.265 | |  |
| **Average spine (T11/T12-L5/S1)** | 40 | -0.31 (-0.68, 0.06) | 0.096 | -0.11 (-0.43, 0.22) | | | 0.515 | -0.02 (-0.07, 0.02) | | | 0.287 | 0.00 (-0.05, 0.05) | | | | | 0.945 | -0.01 (-0.03, 0.02) | | | | 0.624 | 0.01 (-0.03, 0.04) | | | | | | 0.757 | |  |
| **All IVDs** | 280 | -0.25 (-0.47, -0.03) | **0.024** | -0.16 (-0.35, 0.03) | | | 0.096 | -0.03 (-0.06, 0.00) | | | **0.047** | -0.00 (-0.03, 0.03) | | | | | 0.800 | -0.01 (-0.02, 0.01) | | | | 0.301 | 0.01 (-0.01, 0.03) | | | | | | 0.214 | |  |
| **Region 2 IVD T2 (ms)** | | | | |  | | | |  | |  | |  | | |  | | | |  | | |  | | |  | | | | |  |
| **T11/T12** | 40 | -1.05 (-1.84, -0.26) | **0.011** | -0.75 (-1.45, -0.05) | | | **0.038** | -0.05 (-0.16, 0.05) | | | 0.331 | -0.04 (-0.16, 0.08) | | | | | 0.469 | -0.02 (-0.07, 0.03) | | | | 0.488 | 0.03 (-0.05, 0.11) | | | | | | 0.501 | |  |
| **T12/L1** | 40 | -0.43 (-1.27, 0.42) | 0.315 | -0.22 (-0.95, 0.52) | | | 0.556 | 0.03 (-0.08, 0.13) | | | 0.623 | -0.06 (-0.17, 0.06) | | | | | 0.305 | 0.02 (-0.03, 0.07) | | | | 0.363 | 0.04 (-0.04, 0.12) | | | | | | 0.319 | |  |
| **L1/L2** | 40 | 0.10 (-0.81, 1.00) | 0.828 | 0.08 (-0.69, 0.86) | | | 0.827 | 0.06 (-0.04, 0.16) | | | 0.210 | -0.01 (-0.12, 0.10) | | | | | 0.884 | 0.00 (-0.04, 0.05) | | | | 0.853 | 0.02 (-0.06, 0.10) | | | | | | 0.598 | |  |
| **L2/L3** | 40 | -0.14 (-1.14, 0.86) | 0.781 | -0.15 (-1.01, 0.72) | | | 0.732 | 0.05 (-0.06, 0.16) | | | 0.355 | 0.08 (-0.04, 0.20) | | | | | 0.168 | 0.01 (-0.04, 0.06) | | | | 0.747 | 0.02 (-0.07, 0.10) | | | | | | 0.690 | |  |
| **L3/L4** | 40 | -0.84 (-1.96, 0.28) | 0.137 | -0.52 (-1.50, 0.47) | | | 0.294 | -0.09 (-0.21, 0.04) | | | 0.168 | 0.04 (-0.10, 0.18) | | | | | 0.542 | 0.00 (-0.06, 0.06) | | | | 0.972 | 0.06 (-0.03, 0.16) | | | | | | 0.180 | |  |
| **L4/L5** | 40 | -1.59 (-2.82, -0.35) | **0.013** | -0.89 (-2.01, 0.23) | | | 0.115 | -0.03 (-0.18, 0.12) | | | 0.677 | 0.06 (-0.10, 0.23) | | | | | 0.445 | -0.00 (-0.07, 0.06) | | | | 0.915 | 0.02 (-0.09, 0.14) | | | | | | 0.705 | |  |
| **L5/S1** | 40 | -1.77 (-3.03, -0.51) | **0.007** | -0.62 (-1.80, 0.56) | | | 0.293 | -0.13 (-0.29, 0.04) | | | 0.122 | -0.04 (-0.23, 0.14) | | | | | 0.625 | -0.02 (-0.09, 0.06) | | | | 0.666 | -0.05 (-0.17, 0.08) | | | | | | 0.468 | |  |
| **Average spine (T11/T12-L5/S1)** | 40 | -0.81 (-1.59, -0.03) | **0.043** | -0.37 (-1.08, 0.33) | | | 0.288 | -0.00 (-0.09, 0.09) | | | 0.988 | 0.02 (-0.08, 0.12) | | | | | 0.723 | 0.00 (-0.04, 0.04) | | | | 0.968 | 0.01 (-0.05, 0.08) | | | | | | 0.676 | |  |
| **All IVDs** | 280 | -0.82 (-1.22, -0.41) | **<0.001** | -0.44 (-0.79, -0.08) | | | **0.016** | -0.02 (-0.07, 0.03) | | | 0.355 | 0.00 (-0.05, 0.06) | | | | | 0.857 | -0.00 (-0.02, 0.02) | | | | 0.978 | 0.02 (-0.02, 0.06) | | | | | | 0.272 | |  |
| **Region 3 IVD T2 (ms)** | | | | | | |  |  | | |  |  | | | | |  |  | | | |  |  | | | | | |  | |  |
| **T11/T12** | 40 | -1.46 (-2.40, -0.51) | **0.003** | -1.18 (-2.01, -0.35) | | | **0.007** | -0.12 (-0.26, 0.01) | | | 0.063 | -0.05 (-0.20, 0.10) | | | | | 0.535 | -0.04 (-0.10, 0.03) | | | | 0.248 | 0.01 (-0.09, 0.12) | | | | | | 0.827 | |  |
| **T12/L1** | 40 | -0.44 (-1.37, 0.50) | 0.350 | -0.70 (-1.48, 0.08) | | | 0.076 | -0.00 (-0.12, 0.12) | | | 0.984 | -0.04 (-0.16, 0.09) | | | | | 0.572 | 0.02 (-0.03, 0.07) | | | | 0.485 | 0.02 (-0.07, 0.11) | | | | | | 0.680 | |  |
| **L1/L2** | 40 | -0.08 (-1.14, 0.99) | 0.881 | -0.64 (-1.54, 0.26) | | | 0.157 | 0.07 (-0.06, 0.19) | | | 0.288 | 0.03 (-0.11, 0.17) | | | | | 0.690 | -0.01 (-0.07, 0.05) | | | | 0.695 | 0.00 (-0.09, 0.10) | | | | | | 0.962 | |  |
| **L2/L3** | 40 | -0.69 (-1.81, 0.42) | 0.214 | -0.62 (-1.58, 0.34) | | | 0.200 | -0.02 (-0.16, 0.12) | | | 0.778 | 0.08 (-0.07, 0.22) | | | | | 0.303 | -0.02 (-0.08, 0.04) | | | | 0.523 | -0.03 (-0.13, 0.07) | | | | | | 0.541 | |  |
| **L3/L4** | 40 | -1.82 (-3.03, -0.61) | **0.004** | -1.05 (-2.16, 0.06) | | | 0.063 | -0.22 (-0.37, -0.06) | | | 0.008 | -0.02 (-0.21, 0.17) | | | | | 0.831 | -0.05 (-0.12, 0.03) | | | | 0.201 | -0.01 (-0.14, 0.13) | | | | | | 0.928 | |  |
| **L4/L5** | 40 | -1.73 (-3.11, -0.35) | **0.015** | -1.01 (-2.25, 0.24) | | | 0.111 | -0.00 (-0.19, 0.19) | | | 0.987 | 0.06 (-0.15, 0.26) | | | | | 0.582 | -0.04 (-0.12, 0.05) | | | | 0.391 | 0.02 (-0.13, 0.16) | | | | | | 0.807 | |  |
| **L5/S1** | 40 | -1.66 (-3.04, -0.29) | **0.019** | -0.89 (-2.13, 0.36) | | | 0.157 | -0.19 (-0.36, -0.02) | | | **0.030** | -0.05 (-0.25, 0.15) | | | | | 0.594 | -0.02 (-0.10, 0.06) | | | | 0.618 | -0.05 (-0.19, 0.08) | | | | | | 0.442 | |  |
| **Average spine (T11/T12-L5/S1)** | 40 | -1.13 (-1.93, -0.33) | **0.007** | -0.81 (-1.53, -0.09) | | | **0.028** | -0.04 (-0.15, 0.07) | | | 0.476 | 0.02 (-0.10, 0.14) | | | | | 0.710 | -0.02 (-0.07, 0.03) | | | | 0.413 | -0.01 (-0.09, 0.08) | | | | | | 0.874 | |  |
| **All IVDs** | 280 | -1.13 (-1.58, -0.67) | **<0.001** | -0.87 (-1.26, -0.47) | | | **<0.001** | -0.07 (-0.13, -0.01) | | | **0.019** | 0.00 (-0.06, 0.06) | | | | | 0.986 | | -0.02 (-0.05, 0.00) | | | 0.103 | -0.01 (-0.05, 0.04) | | | | 0.797 | | |  |  |
| **Region 4 IVD T2 (ms)** | | | | | | | | | |  | | |  | | |  | | | | | |  | | |  | | | | | |  |
| **T11/T12** | 40 | -1.46 (-2.58, -0.35) | **0.011** | -1.19 (-2.16, -0.22) | | | **0.018** | -0.12 (-0.27, 0.03) | | | 0.116 | 0.00 (-0.17, 0.18) | | | | | 0.973 | -0.02 (-0.09, 0.05) | | | | 0.506 | -0.03 (-0.15, 0.09) | | | | | | 0.630 | |  |
| **T12/L1** | 40 | -0.42 (-1.65, 0.81) | 0.494 | -1.10 (-2.11, -0.09) | | | **0.033** | -0.07 (-0.22, 0.07) | | | 0.314 | -0.04 (-0.21, 0.12) | | | | | 0.604 | 0.02 (-0.05, 0.08) | | | | 0.648 | -0.05 (-0.16, 0.06) | | | | | | 0.396 | |  |
| **L1/L2** | 40 | -0.32 (-1.63, 0.99) | 0.626 | -0.91 (-2.00, 0.19) | | | 0.101 | -0.05 (-0.21, 0.11) | | | 0.493 | -0.03 (-0.21, 0.14) | | | | | 0.696 | -0.01 (-0.08, 0.06) | | | | 0.814 | -0.07 (-0.19, 0.05) | | | | | | 0.253 | |  |
| **L2/L3** | 40 | -0.58 (-1.72, 0.55) | 0.306 | -0.49 (-1.47, 0.49) | | | 0.313 | -0.10 (-0.24, 0.04) | | | 0.155 | -0.01 (-0.17, 0.15) | | | | | 0.879 | -0.01 (-0.08, 0.05) | | | | 0.650 | -0.06 (-0.17, 0.05) | | | | | | 0.299 | |  |
| **L3/L4** | 40 | -1.15 (-2.43, 0.14) | 0.078 | -0.78 (-1.91, 0.34) | | | 0.168 | -0.24 (-0.39, -0.08) | | | 0.004 | -0.08 (-0.27, 0.11) | | | | | 0.390 | -0.04 (-0.12, 0.03) | | | | 0.259 | -0.04 (-0.17, 0.09) | | | | | | 0.534 | |  |
| **L4/L5** | 40 | -1.28 (-2.34, -0.22) | **0.019** | -0.61 (-1.58, 0.35) | | | 0.206 | -0.04 (-0.18, 0.10) | | | 0.549 | 0.00 (-0.15, 0.16) | | | | | 0.965 | -0.05 (-0.11, 0.01) | | | | 0.129 | -0.01 (-0.12, 0.10) | | | | | | 0.830 | |  |
| **L5/S1** | 40 | -0.63 (-1.73, 0.47) | 0.252 | -0.78 (-1.71, 0.15) | | | 0.096 | -0.12 (-0.26, 0.02) | | | 0.079 | -0.04 (-0.19, 0.12) | | | | | 0.630 | -0.02 (-0.08, 0.05) | | | | 0.553 | -0.05 (-0.16, 0.06) | | | | | | 0.351 | |  |
| **Average spine (T11/T12-L5/S1)** | 40 | -0.81 (-1.71, 0.10) | **0.080** | -0.75 (-1.53, 0.02) | | | 0.057 | -0.07 (-0.19, 0.05) | | | 0.225 | -0.01 (-0.15, 0.12) | | | | | 0.845 | -0.02 (-0.07, 0.04) | | | | 0.521 | -0.04 (-0.13, 0.05) | | | | | | 0.388 | |  |
| **All IVDs** | 280 | -0.83 (-1.29, -0.38) | **<0.001** | -0.84 (-1.23, -0.45) | | | **<0.001** | -0.11 (-0.16, -0.05) | | | **<0.001** | -0.03 (-0.09, 0.04) | | | | | 0.377 | -0.02 (-0.05, 0.01) | | | | 0.129 | -0.04 (-0.09, 0.00) | | | | | | 0.052 | |  |
| **Region 5 IVD T2 (ms)** | | | | | |  | | |  | | | | |  |  | | | | | |  | | |  | | | |  | | | |
| **T11/T12** | 40 | -0.53 (-1.22, 0.15) | 0.123 | -0.48 (-1.07, 0.11) | | | 0.106 | -0.03 (-0.12, 0.06) | | | 0.492 | 0.06 (-0.04, 0.16) | | | | | 0.234 | -0.02 (-0.06, 0.02) | | | | 0.363 | -0.03 (-0.10, 0.04) | | | | | | 0.448 | |  |
| **T12/L1** | 40 | 0.09 (-0.94, 1.11) | 0.866 | -0.27 (-1.15, 0.61) | | | 0.537 | -0.05 (-0.18, 0.08) | | | 0.422 | -0.03 (-0.17, 0.11) | | | | | 0.623 | -0.02 (-0.08, 0.04) | | | | 0.447 | -0.10 (-0.19, -0.01) | | | | | | **0.032** | |  |
| **L1/L2** | 40 | 0.04 (-0.79, 0.86) | 0.930 | -0.21 (-0.92, 0.50) | | | 0.559 | -0.10 (-0.20, 0.00) | | | **0.049** | -0.02 (-0.14, 0.09) | | | | | 0.665 | -0.01 (-0.05, 0.04) | | | | 0.769 | -0.07 (-0.14, 0.01) | | | | | | 0.079 | |  |
| **L2/L3** | 40 | -0.52 (-1.18, 0.14) | 0.120 | -0.29 (-0.87, 0.29) | | | 0.320 | -0.09 (-0.17, -0.01) | | | **0.036** | -0.04 (-0.13, 0.06) | | | | | 0.459 | -0.01 (-0.05, 0.03) | | | | 0.623 | -0.04 (-0.11, 0.02) | | | | | | 0.193 | |  |
| **L3/L4** | 40 | -0.35 (-0.84, 0.13) | 0.151 | -0.14 (-0.57, 0.29) | | | 0.504 | -0.03 (-0.09, 0.03) | | | 0.297 | -0.05 (-0.12, 0.02) | | | | | 0.136 | -0.01 (-0.03, 0.02) | | | | 0.649 | -0.02 (-0.07, 0.02) | | | | | | 0.306 | |  |
| **L4/L5** | 40 | -0.08 (-0.48, 0.32) | 0.687 | 0.10 (-0.25, 0.44) | | | 0.574 | -0.01 (-0.06, 0.04) | | | 0.779 | -0.03 (-0.09, 0.02) | | | | | 0.195 | -0.01 (-0.03, 0.01) | | | | 0.343 | -0.02 (-0.06, 0.01) | | | | | | 0.222 | |  |
| **L5/S1** | 40 | 0.16 (-0.54, 0.85) | 0.654 | -0.21 (-0.81, 0.40) | | | 0.494 | 0.03 (-0.05, 0.11) | | | 0.471 | -0.04 (-0.13, 0.05) | | | | | 0.386 | 0.00 (-0.03, 0.04) | | | | 0.844 | -0.03 (-0.10, 0.03) | | | | | | 0.271 | |  |
| **Average spine (T11/T12-L5/S1)** | 40 | -0.27 (-0.74, 0.20) | 0.259 | -0.17 (-0.58, 0.24) | | | 0.401 | -0.04 (-0.09, 0.02) | | | 0.218 | -0.03 (-0.10, 0.03) | | | | | 0.284 | -0.01 (-0.03, 0.02) | | | | 0.547 | -0.04 (-0.08, 0.01) | | | | | | 0.090 | |  |
| **All IVDs** | 280 | -0.17 (-0.46, 0.11) | 0.237 | -0.21 (-0.46, 0.03) | | | 0.089 | -0.04 (-0.07, -0.00) | | | **0.028** | -0.02 (-0.06, 0.02) | | | | | 0.256 | -0.01 (-0.03, 0.01) | | | | 0.222 | -0.05 (-0.07, -0.02) | | | | | | **0.001** | |  |

Data are linear regression of independent variable total BPAQ score and dependent variable magnetic resonance IVD T2 across all participants, males and females. Beta coefficient (β**)** indicate the relationship that moves in the same direction and variance as 95% confidence interval (95%CI). Pfirrmann grade is reverse scale where negative values indicate less degeneration. BPAQ: Bone-specific Physical Activity Questionnaire; Sex: biological sex; BMI: body mass index, IVD: Intervertebral disc; T2: time constant of the rate of proton relaxation in response to magnetization wither higher values indicating greater hydration.
